# Heparan Sulfate Chain‐Conjugated Laminin‐E8 Fragments Advance Paraxial Mesodermal Differentiation Followed by High Myogenic Induction from hiPSCs

**DOI:** 10.1002/advs.202308306

**Published:** 2024-04-29

**Authors:** Mingming Zhao, Yukimasa Taniguchi, Chisei Shimono, Tatsuya Jonouchi, Yushen Cheng, Yasuhiro Shimizu, Minas Nalbandian, Takuya Yamamoto, Masato Nakagawa, Kiyotoshi Sekiguchi, Hidetoshi Sakurai

**Affiliations:** ^1^ Department of Clinical Application Center for iPS Cell Research and Application (CiRA) Kyoto University 53 Shogoin‐Kawahara‐cho, Sakyo‐ku Kyoto 606‐8507 Japan; ^2^ Center for Medical Epigenetics School of Basic Medical Sciences Chongqing Medical University 1 Yixueyuan Road, Yuzhong District Chongqing 400016 China; ^3^ Division of Matrixome Research and Application Institute for Protein Research Osaka University 3‐2 Yamadaoka, Suita Osaka 565‐0871 Japan; ^4^ Department of Life Science Frontiers Center for iPS Cell Research and Application (CiRA) Kyoto University 53 Shogoin‐Kawahara‐cho, Sakyo‐ku Kyoto 606‐8507 Japan

**Keywords:** fibroblast growth factor signaling, heparan sulfate, human pluripotent stem cells, new generation laminin fragments, paraxial mesoderm

## Abstract

Human‐induced pluripotent stem cells (hiPSCs) have great therapeutic potential. The cell source differentiated from hiPSCs requires xeno‐free and robust methods for lineage‐specific differentiation. Here, a system is described for differentiating hiPSCs on new generation laminin fragments (NGLFs), a recombinant form of a laminin E8 fragment conjugated to the heparan sulfate chains (HS) attachment domain of perlecan. Using NGLFs, hiPSCs are highly promoted to direct differentiation into a paraxial mesoderm state with high‐efficiency muscle lineage generation. HS conjugation to the C‐terminus of Laminin E8 fragments brings fibroblast growth factors (FGFs) bound to the HS close to the cell surface of hiPSCs, thereby facilitating stronger FGF signaling pathways stimulation and initiating HOX gene expression, which triggers the paraxial mesoderm differentiation of hiPSCs. This highly efficient differentiation system can provide a roadmap for paraxial mesoderm development and an infinite source of myocytes and muscle stem cells for disease modeling and regenerative medicine.

## Graphical abstract

This study describes the new generation laminin fragments, a recombinant form of a laminin E8 fragment conjugated to the heparan sulfate chains. Taking advantage of the new generation laminin fragments, authors established a highly efficient and xeno‐free protocol for paraxial mesoderm differentiation of hiPSCs followed by high myogenic induction, providing an attractive cell source for disease modeling and cell therapy.

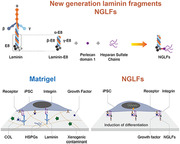

## 1 Introduction

Human induced pluripotent stem cells (hiPSCs) can typically proliferate and self‐renew indefinitely in vitro and differentiate into specialized cell types that have great potential for a wide range of biomedical applications in regenerative medicine and as tools for human disease modeling.^[^ ^1^, ^2^ ^]^ Multiple reports have successfully established a lineage‐specific differentiation protocol for generating myocytes and muscle stem cells (MuSCs) from hiPSCs, showing regeneration potential in severe and progressive muscle dystrophy.^[^ ^3^, ^4^, ^5^, ^6^ ^]^ However, these differentiation protocols still show lower efficiency and require an animal‐derived Matrigel. For clinical applications, establishing a highly efficient and xeno‐free (animal‐derived substance‐free) environment for inducing hiPSC differentiation is therefore necessary.

**Figure 1:**
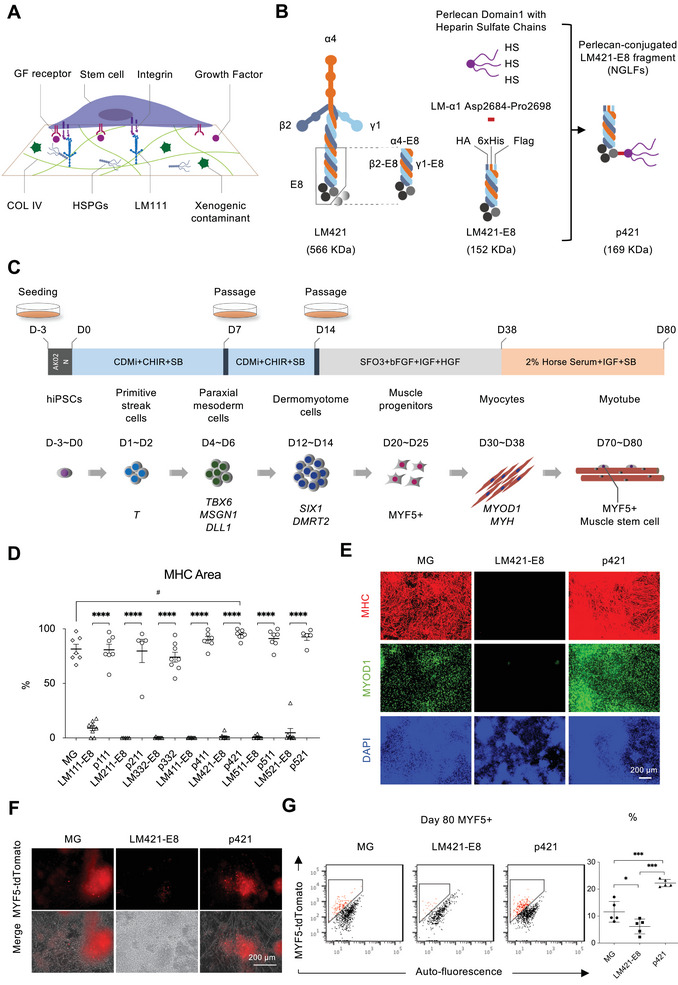
Screening NGLFs to optimize myocytes induction from hiPSCs. a) Schematic representations of stem cell growth in MG, which is extracted from the Engelbreth‐Holm‐Swarm (EHS) mouse sarcoma and contains 60% laminin111 (LM111), 30% collagen IV (COL IV), and heparan sulfate proteoglycans (HSPGs). MG also contains growth factors (GFs) and xenogenic contaminants leading to undesirable effects in stem cell differentiation. b) Schematic representations of LM421, LM421‐E8, and p421. The structure of the E8 fragment is indicated in the grey box in LM421. For protein purification, three types of peptide tags (6xHis, HA (human influenza hemagglutinin), or FLAG) were added to the N‐termini of the laminin α4Ε8, β2Ε8, and γ1E8 chains. To add HS to LM421‐E8, the perlecan D1 domain carrying three heparan sulfate (HS) chains was attached to the C‐terminus of the laminin α4Ε8 chain. The D1 domain of Perlecan was attached to the C‐terminus of the LMα4‐E8 fragment through a 15 amino acid segment derived from the linker region of the laminin α1 chain (Figure S11, Supporting Information). The molecular weights of the individual proteins are shown in parentheses. c) Schematic representation of stepwise induction and differentiation of skeletal muscle from hiPSCs cultured in Stemfit (AK02N) for three days. The medium was changed to CDMi supplemented with SB431542 (SB) (5 µm) and CHIR99021 (CHIR) (10 µm) and then cultured for 14 days. Cells were passaged on days 7 and 14 as single cells. Next, the cells were cultured on SFO3 (0.2% (w/v) BSA, 0.1 mm 2‐ME) supplemented with bFGF (10 µm), IGF (10 µm) and HGF (10 µm). On day 38, the medium was changed to DMEM supplemented with 2% Horse serum, 5 µm SB 431542, and 10 ng mL^−1^ IGF‐1 until day 80. d) Quantification of % area covered by myocyte (MHC+) after 38 days of differentiation (Figure S1, Supporting Information). Error bars, mean ± SD, n ≥ 5. P‐values were obtained using a one‐way ANOVA with Tukey's multiple comparisons test. ^****^ *P* < 0.0001; ^#^ *P* < 0.05. e) MHC, MYOD1, and DAPI staining of MYF5‐Tdtomato hiPSC line derived myocytes (day 38) in MG, LM421‐E8, and p421. f) Representative tdTomato images of live MYF5‐tdTomato cells and myotubes at day 80 of differentiation. g) Flow cytometric evaluation of MYF5+ cell population at 80 days of differentiation in MG, LM421‐E8, and p421. Quantification of % of MYF5+ cell population at day 80 of differentiation. Error bars, mean ± SD, n = 5. P‐values were obtained using a one‐way ANOVA with Tukey's multiple comparisons test. ^*^ *P* < 0.05, ^***^ *P* < 0.001.

Matrigel (MG), which is extracted from Engelbreth‐Holm‐Swarm mouse tumors, has been commonly used for the maintenance of undifferentiated hiPSCs ^[^ ^7^ ^]^ and hiPSC differentiation.^[^ ^3^, ^4^, ^5^, ^6^, ^8^ ^]^ MG primarily consists of 60% laminin, 30% collagen type IV, 8% entactin, and 2% perlecan, closely resembling the complex extracellular matrix of the basement membrane and supporting for hiPSCs culture (**Figure** **1**A).^[^ ^9^, ^10^, ^11^ ^]^ However, MG also contains an ill‐defined composition of proteases and animal‐derived factors, leading to batch‐to‐batch variability and experimental uncertainty.^[^ ^9^ ^]^ The limitations of MG motivated us to search for a highly tunable recombinant scaffold for xeno‐free hiPSC differentiation.

Laminins are a family of glycoproteins that are present in the basement membrane. All laminins are large heterotrimeric glycoproteins composed of α, β, and γ chains that assemble into cross‐shaped structures (Figure 1B).^[^ ^12^ ^]^ They are named according to their chain composition; for example, laminin 421 consists of α4, β2, and γ1 chains. Recently, recombinant laminin E8 fragments (LM‐E8s) (Figure 1B), serving as a functionally minimal form,^[^ ^12^ ^]^ were used as xeno‐free coating substrates for in vitro hiPSC cultures in numerous studies.^[^ ^13^, ^14^ ^]^ However, our results indicated that LM‐E8s did not support myogenic differentiation, indicating that components other than laminin in MG induce myogenic differentiation of hiPSCs.

MG contains perlecan, a heparan sulfate proteoglycan (HSPG) with a core protein of more than 4000 amino acids, to which three heparan sulfate chains (HS) are attached (Figure 1A).^[^ ^9^ ^]^ Perlecan is a multifunctional HSPG that regulates hiPSC differentiation by interacting with several growth factors, cytokines, and other signaling molecules.^[^ ^15^ ^]^ However, full‐length perlecan has five distinct structural domains with a molecular weight of 470 kDa, and the mass production of recombinant perlecan is very difficult. Therefore, we combined domain 1 (D1) of perlecan with HS to the C‐terminus of the LM‐E8 fragment and named this newly designed recombinant protein new generation laminin fragments (NGLFs) (Figure 1B).

In this study, we found that NGLFs robustly induced myogenic differentiation via highly efficient promotion of the paraxial mesoderm lineage. Conjugation of HS to the C‐terminus of LM421‐E8s brought the HS‐bound fibroblast growth factors (FGFs) close to the fibroblast growth factor receptor (FGFR) on the cell surface, strongly stimulating the FGF signaling pathway during the pre‐culture and early differentiation stages, thereby regulating paraxial mesoderm differentiation of hiPSCs. NGFLs induced the expression of HOX genes during the pre‐culture stage, which primed hiPSCs to differentiate into the paraxial mesoderm lineages. Using these xeno‐free NGLFs we established a highly efficient method for paraxial mesoderm differentiation and, subsequently myogenic differentiation, providing an attractive cell source for disease modeling and clinical application.

## 2 Results

### 2.1 Screening NGLFs in Myocytes Induction from hiPSCs

Previously, we reported a stepwise protocol for myogenic differentiation of hiPSCs following skeletal muscle development (Figure 1C).^[^ ^6^ ^]^ In this system, hiPSCs passed three key embryonic stages: the primitive streak (PS) stage (day 1–2), the paraxial mesoderm (PM) stage (day 4–6), and the dermomyotome (DM) stage (day 12–14). The dermomyotome gives rise to skeletal muscle progenitors, which transiently express MYF5 and rapidly commit to myogenesis to myocytes expressing MYOD1 and myosin heavy chain (MHC) (day 25–38). Then, cells mature to form myotubes and MuSCs (at day 70–80) (Figure 1C).^[^ ^4^, ^6^ ^]^ However, these cells were cultured on an animal‐derived MG material, which cannot be used for clinical applications (Figure 1A). To replace the MG in this system, we first tested different LM‐E8s (LM111‐E8, LM211‐E8, LM332‐E8, LM411‐E8, LM421‐E8, LM511‐E8, and LM521‐E8) as candidate scaffolds for myogenic induction. Unfortunately, each LM‐E8 provided significantly lower efficiency in myogenic differentiation compared to MG (Figure 1A–D; Figure S1A, Supporting Information). Next, we screened the NGLFs, the LM‐E8 fragments with the HS‐attached D1 of perlecan, to induce myogenic development in vitro (Figure 1C). The NGLFs stimulated robust myogenic differentiation, as indicated by immunostaining of MYOD1 and MHC (Figure 1E; Figure S1B, Supporting Information). Of the NGLF isoforms, p421 showed the highest MHC area on day 38, indicating more homogeneous myocyte formation (Figure 1D; Figure S1B, Supporting Information). To observe the formation of MuSCs, we used the MYF5‐tdTomato reporter line in 201B7 hiPSCs,^[^ ^4^, ^6^ ^]^ which could report MuSCs around day 80. Notably, compared to MG, p421 significantly improved myocyte and MuSC development from hiPSCs (Figure 1E–G), prompting us to select p421 as a candidate to establish a highly efficient protocol for myocyte and MuSC induction from hiPSCs.

### 2.2 P421 Advanced Paraxial Mesodermal Differentiation Followed by High Myogenic Induction

**Figure 2:**
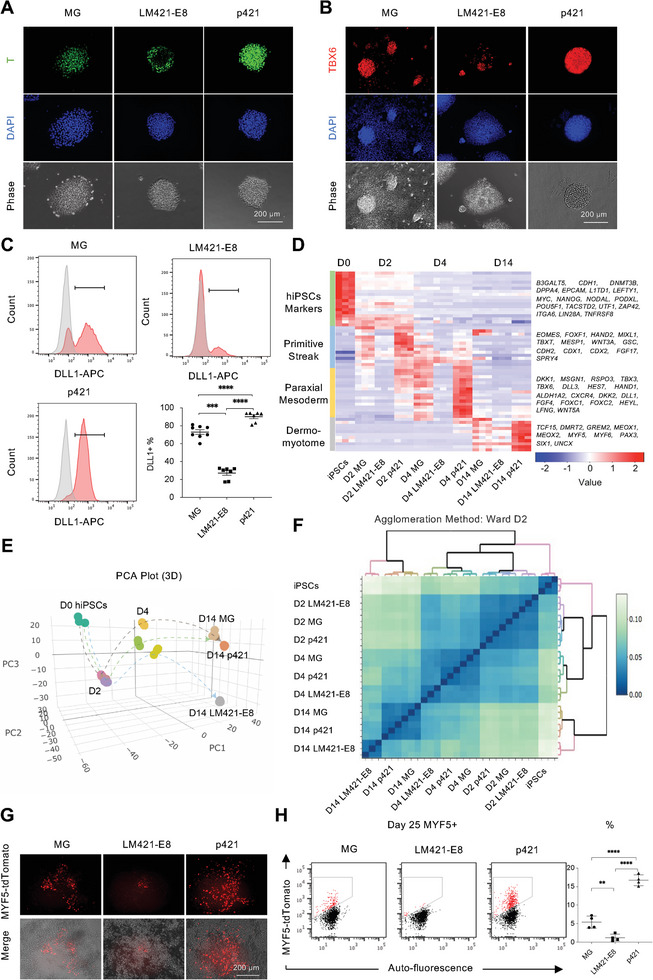
p421 supports efficient myogenic differentiation from hiPSCs. a) BRACHYURY (T) and DAPI staining of the hiPSC‐derived primitive streak (day 2) in MG, LM421‐E8, and p421; scale bar, 200 µm. b) TBX6 and DAPI staining of hiPSC‐derived paraxial mesoderm (day 4) in MG, LM421‐E8, and p421; scale bar, 100 µm. c) Flow cytometric evaluation of DLL1+ cell population on day 4 of differentiation in MG, LM421‐E8, and p421. Error bars, mean ± SD. n ≥ 7. *P*‐values were obtained using a one‐way ANOVA with Tukey's multiple comparison test. ^***^ *P* < 0.001, ^****^ *P* < 0.0001. d) Heat map of normalized gene expression levels for marker genes of hiPSCs, primitive streak (PS), paraxial mesoderm (PM), and dermomyotome (DM) (n = 3). e) Principal component analysis (PCA) plots of cells on days 0 (D0), 2 (D2), 4 (D4), and 14 (D14) (n = 3). f) Correlation matrix heatmap with hierarchical clustering analysis among cells on D0, D2, D4, and D14 (n = 3). g) Representative tdTomato images of live MYF5‐tdTomato cells on day 25 of differentiation. Flow cytometric evaluation of the MYF5+ cell population at 25 days of differentiation in MG, LM421‐E8, and p421. h) Quantification of the percentage of the MYF5+ cell population on day 25 of differentiation. Error bars, mean ± SD, n = 4. *P*‐values were obtained using a one‐way ANOVA with Tukey's multiple comparison test. ^**^ *P* < 0.01, ^****^ *P* < 0.0001.

To ensure that p421 is comparable to MG in myogenic differentiation, we assessed its capacity at all differentiation stages. The marker gene expression of *T* at the PS stage (Figure S2A, Supporting Information), *TBX6* and *MSGN1* at the PM stage (Figure S2B,C, Supporting Information), and *SIX1* and *DMRT2* at the DM stage (Figure S2D,E, Supporting Information)^[^ ^6^ ^]^ was significantly higher in NGLFs than in LM‐E8s. Among the NGLFs studied, p421 showed the highest marker gene expression at each stage, which was significantly higher than that seen in MG. Therefore, we selected p421 and LM421‐E8 to analyze the efficacy of NGLFs for myogenic induction from hiPSCs. Consistent with the gene expression results, T and TBX6 were highly expressed in cells cultured on p421 at the protein level (**Figure** **2**A,B). p421 provided more than 90% of the PM cell population, which was indicated by the PM surface marker DLL1 (Figure 2C). RNA‐sequencing (RNA‐seq) analysis of samples cultured on MG, LM421‐E8, and p421 revealed that in each stage before DM formation, transcriptional markers of each stage were robustly expressed in cells cultured on p421^[^ ^16^ ^]^ (Figure 2D). Transcriptome analysis during hiPSC differentiation showed that p421 clustered closer to the MG but farther from LM421‐E8 (Figure 2E,F). Moreover, p421 significantly increased the expression of marker genes during PS on day 2 and PM formation on day 4 (Figure S3A–F, Supporting Information). In contrast, compared with p421, LM421‐E8 highly induced the expression of undifferentiated marker (*SOX2*) on days 2 and 4 (Figure S3E, Supporting Information). Gene ontology (GO) analysis showed enrichment in the gene related to segmentation and somite development in p421 and enrichment of neurogenesis in LM421‐E8 (Figure S3C–H, Supporting Information). Around day 25, MYF5, a marker of muscle progenitors, was transiently expressed. We detected a much higher MYF5+ cell population in p421 cells than in MG or LM421‐E8 cells (Figure 2G,H). Subsequently, higher myocyte and MuSC populations were detected on p421 on days 38 and 80 (Figure 1F,G). We thus showed that p421 can robustly improve PM development and give rise to muscle lineage cells.

### 2.3 HS of p421 Effects in the Stage of Paraxial Mesoderm Formation

**Figure 3:**
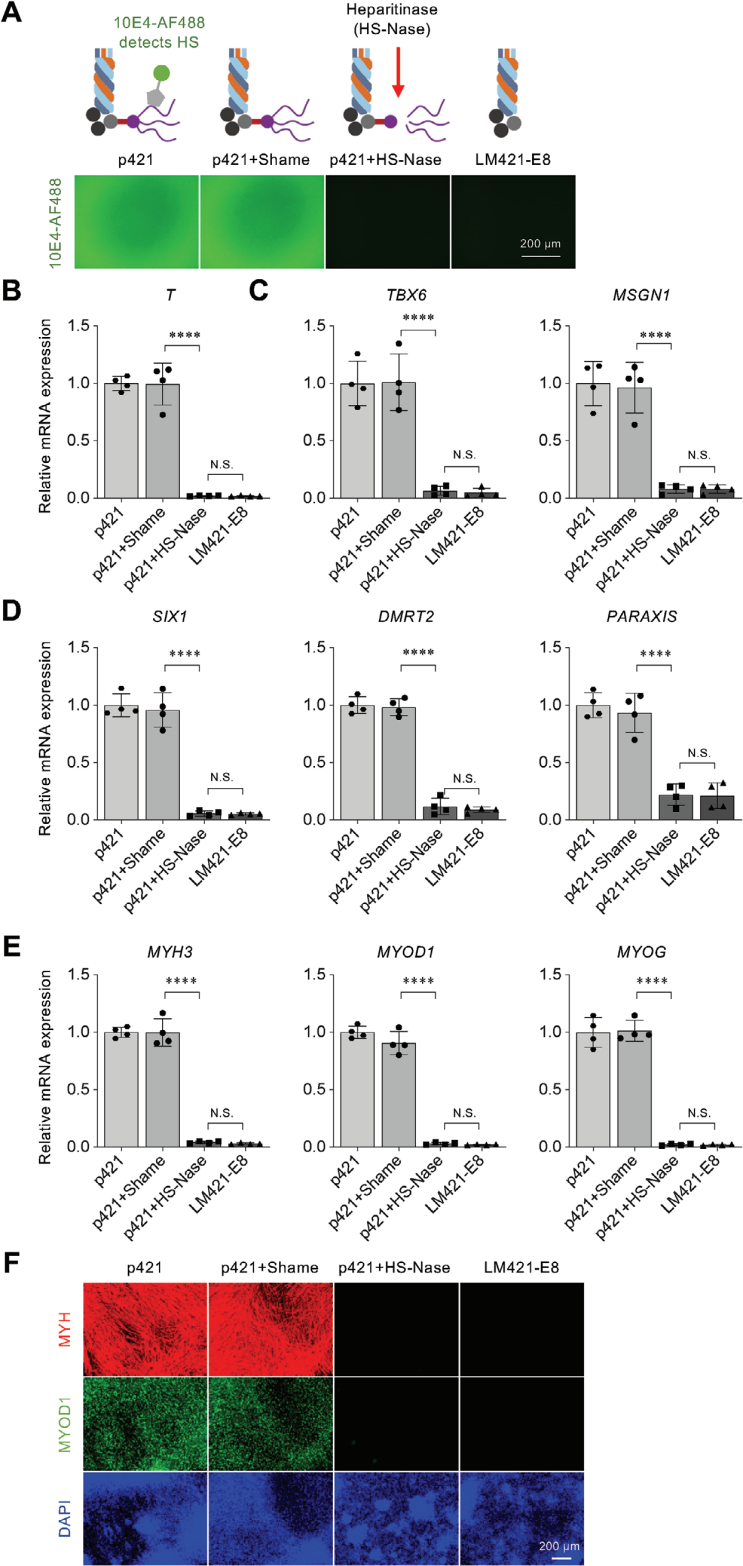
p421 induces hiPSCs differentiation through HS chains. a) Schematic illustration of the degradation of heparan sulfate chains (HS) of p421 in a culture dish pre‐coated with p421. The dishes were then treated with a reaction buffer containing heparinase (p421+shame) or heparinase with a reaction buffer (HS‐Nase). p421 and LM421‐E8 coated dishes were used as positive and negative controls, respectively. The 10E4 antibody recognizes a common epitope on HS. Staining with 10E4‐AF488 revealed heparinase‐degrading HS in the p421‐coated dish. b–e) qRT‐PCR comparing the expression levels of *T* (day2), *TBX6* (day4), *MSGN1* (day4), *SIX1* (day14), *DMRT2* (day14), *PARAXIS* (day14), *MYH3* (day38) and *MYOD1* (day38) in differentiated hiPSCs cultured on p421, p421+Shame, p421+HS‐Nase, and LM421‐E8. Error bars, mean ± SD, n = 4. *P*‐values were obtained using a one‐way ANOVA with Tukey's multiple comparison test. ^****^ *P* < 0.0001; N.S.: not significant. f) MHC, MYOD1, and DAPI staining of hiPSC‐derived myocytes (day 38) in p421, p421+Shame, p421+HS‐Nase, and LM421‐E8 cells; scale bar, 200 µm.

HS is an unbranched polysaccharide chain composed of repeating disaccharide units that are highly hydrophilic and can bind growth factors. They form a growth factor reservoir, protecting growth factors from degradation, and regulating their transport and accessibility.^[^ ^17^, ^18^ ^]^ To determine whether HS is the functional component of p421 that regulates myogenic differentiation, HS chains were degraded on p421‐coated dishes using heparinase treatment (**Figure** **3**A). Degradation of HS eliminated the differentiation‐promoting activity of p421, indicated by a decrease in marker gene expression of PS (*T*), PM (*TBX6* and *MSGN1*), DM (*SIX1*, *DMRT2*, and *PARAXIS*), and myocytes (*MYH3*, *MYOD1*, and *MYOG*) (Figure 3B–E) and the disruption of myocyte induction (Figure 3F). The protein levels of T and TBX6 were similar to the gene expression levels (Figure S7A,B, Supporting Information). These results suggest the HS of p421 is a major component for regulating the differentiation from hiPSCs.

**Figure 4:**
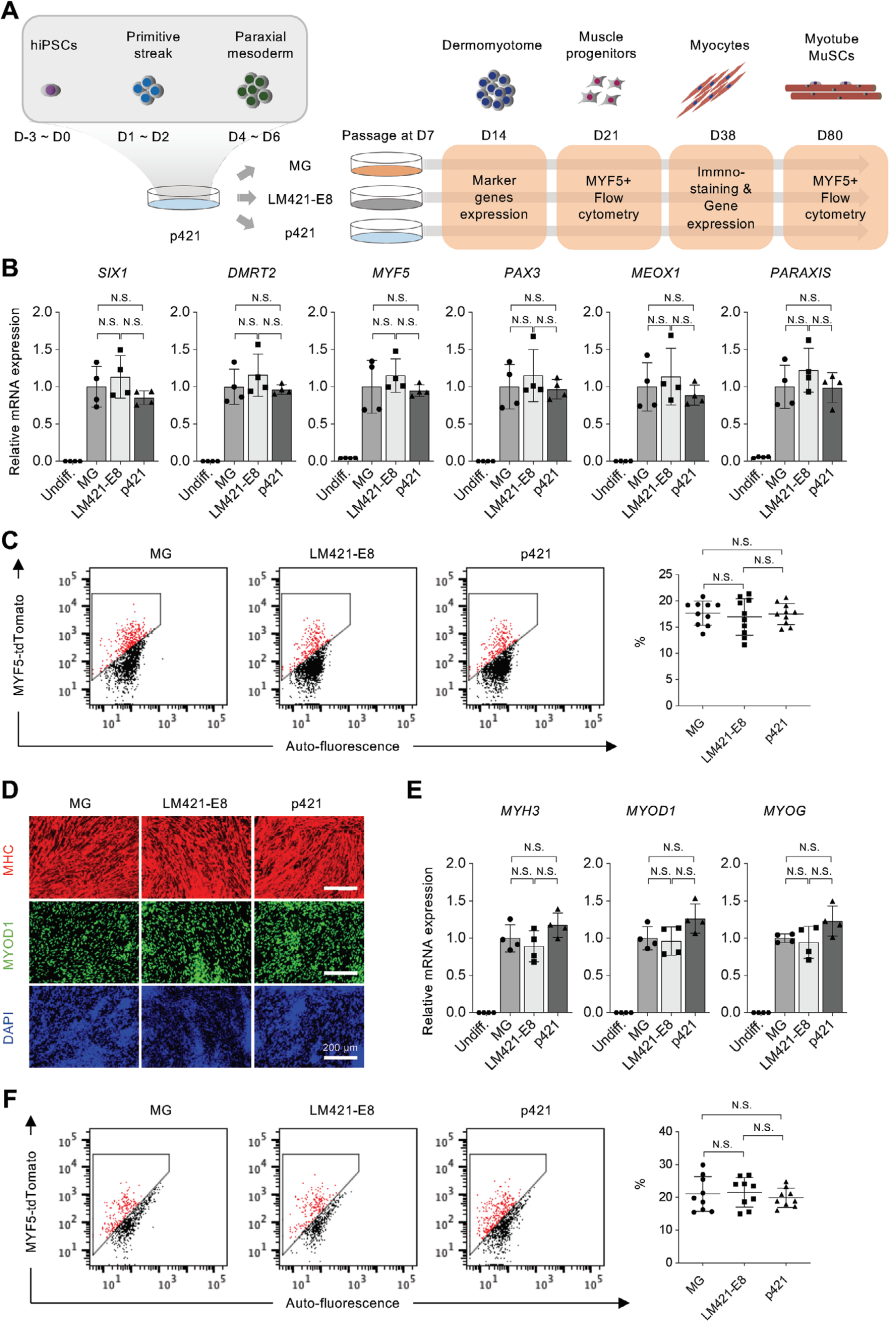
The effects of p421 in hiPSCs differentiation are confined to the stage of paraxial mesoderm formation. a) Schematic illustration of detecting the functional period of p421 in step‐wised myogenic differentiation from hiPSCs. The hiPSCs were cultured on a p421‐coated dish from days ‐3–7 to induce primitive streak and paraxial mesoderm formation. On day 7, p421 cultured cells were passaged to MG, LM421‐E8, or p421 to induce subsequent differentiation. Dermomyotome markers on day 14, skeletal muscle progenitor cell markers on day 21, myocyte markers on day 38, and muscle stem cell population were detected respectively. b) qRT‐PCR analysis comparing the dermomyotome marker genes (day 14) expression level of *SIX1*, *DMRT2*, *MYF5, PAX3, MEOX1*, and *PARAXIS* in MG, LM421‐E8s, or p421. Error bars, mean ± SD, n = 4. *P*‐values were obtained using a one‐way ANOVA with Tukey's multiple comparisons test. N.S.: not significant. c) Flow cytometric evaluation of MYF5+ cells population at 25 days of differentiation in MG, LM421‐E8, and p421. Quantification of % of MYF5+ cell population at day 25 of differentiation (right) in MG, LM421‐E8, and p421. Error bars, mean ± SD, n = 10. *P*‐values were obtained using a one‐way ANOVA with Tukey's multiple comparison test. N.S.: not significant. d) MHC, MYOD1, and DAPI staining of hiPSCs derived myocytes (day 38) in MG, LM421‐E8, and p421. e) qRT‐PCR analysis comparing the myocyte marker genes (day 38) expression level of *MYH3*, *MYOD1*, and *MYOG* in MG, LM421‐E8s, and p421. Error bars, mean ± SD, n = 4. N.S.: not significant. f) Flow cytometric evaluation of MYF5+ cells population at 80 days of differentiation in MG, LM421‐E8, and p421. Quantification of % of MYF5+ cell population at day 80 of differentiation (right) in MG, LM421‐E8, and p421. Error bars, mean ± SD, n = 9. *P*‐values were obtained using a one‐way ANOVA with Tukey's multiple comparison test. N.S.: not significant.

Next, we examined the working stage of HS by using an 80‐day stepwise protocol. Since cells needed to pass at days 7 and 14 in this myogenic differentiation system,^[^ ^6^ ^]^ we cultured cells on p421‐coated dishes until paraxial mesoderm formation on day 7. Next, the cells were passaged to MG, LM421‐E8, or p421‐coated dishes (**Figure** **4**A). Passaged cells cultured on MG, LM421‐E8, or p421 showed the same myogenic differentiation capacity, as indicated by the marker gene expression of the dermomyotome (*SIX1*, *DMRT2*, *MYF5*, *PAX3*, *MEOX1*, and *PARAXIS*) at day 14 (Figure 4B), muscle progenitor population at day 25 (Figure 4C), and myocyte markers (*MYH3*, *MYOD1*, and *MYOG*) on day 38 (Figure 4E). Consistent with the gene expression results, MG, LM421‐E8, and p421 induced a similar population of myocytes on day 38 and MuSCs on day 80 (Figure 4D–F).

Taken together, these results indicate that the conjugated HS in p421 regulates hiPSCs differentiation in the PM formation stage. Before the first passage on day 7, cells were cultured on a coated matrix for two stages: the pre‐culture stage (day ‐3–0) and the early differentiation stage (day 0–7) (Figure 1C). To clarify the working stage and the effects of HS on p421, we sought to investigate the effects of HS in these two stages.

### 2.4 HS Induces Paraxial Mesoderm Formation by Activating the FGF Signaling Pathway

**Figure 5:**
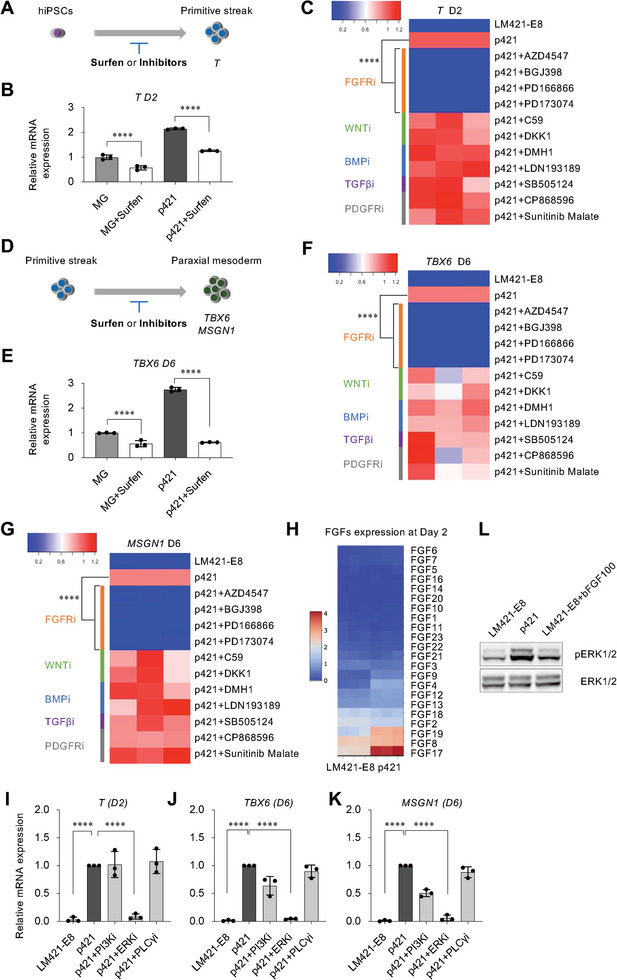
HS regulating FGF signaling in the paraxial mesoderm differentiation from hiPSCs. a) Schematic illustration of treating cells with surfen or inhibitors from D0–D2. b) After stimulating differentiation, cells cultured on p421 were incubated with surfen from D0–D2. qRT‐PCR analysis comparing the expression level of *T* after treating with surfen. Error bars, mean ± SD, n = 3. c) Cells cultured on p421 were incubated with inhibitors from D0–D2. FGFR inhibitors: AZD4547 (1 µM), BGJ398 (1 µm), PD166866 (1 µm), and PD173074 (1 µm); Wnt inhibitors: Wnt‐C59 (1 µm) and DKK1 (1 µm); BMP inhibitors: DMH1 (1 µm) and LDN193189 (1 µm); TGFβ inhibitor: SB505124 (1 µm); PDGF inhibitors: CP868596 (1 µm) and Sunitinib Malate (1 µm). qRT RT‐PCR analysis comparing the expression level of *T* on D2 after treatment with inhibitors. Error bars, mean ± SD, n = 3. d) Schematic illustration of treating cells with surfen or inhibitors from D2–D6. e) Cells cultured on p421 were incubated with surfen from D2–D6. qRT‐PCR analysis comparing the expression level *TBX6* on D6 after treating with surfen. Error bars, mean ± SD, n = 3. f,g) Cells cultured on p421 were incubated with inhibitors from D2–D6. qRT‐PCR analysis comparing the expression level *TBX6* and *MSGN1* on D6 after treatment with inhibitors. Error bars, mean ± SD, n = 3. h) Heatmap of FGF expression in cells cultured on LM421‐E8 or p421 on day 2 of differentiation. i,j) Cells cultured on p421 were incubated with FGFR downstream inhibitors, PIK inhibitor (PIK90, 1 µm)), ERK inhibitor (PD0325901, 1 µm), or PLC inhibitor (U73122, 10 µm) from D0–D2 or from D2‐D6. qRT‐PCR analysis comparing the expression level of *T* on D2 after treatment with inhibitors. Error bars, mean ± SD, n = 3. j,k) Cells cultured on p421 were incubated with FGFR downstream inhibitors from D2–D6. qRT‐PCR analysis comparing the expression level *TBX6* and *MSGN1* on D6 after treatment with inhibitors. Error bars, mean ± SD, n = 3. *P*‐values were obtained using a one‐way ANOVA with Tukey's multiple comparisons test. ^*^ *P* < 0.05, ^**^ *P* < 0.01, ^***^ *P* < 0.001, ^****^ *P* < 0.0001. l) Western blot analysis of phosphorylation‐p44/42 (Thr202/Tyr204) of ERK (pERK) at day 2 of the differentiation with the conditions of LM421‐E8, p421 and LM421‐E8 with bFGF (100 ng mL^−1^).

To confirm the effects of HS on PM formation, we examined whether surfen (bis‐2‐methyl‐4‐amino‐quinolyl‐6‐carbamide), a small molecule antagonist of heparan sulfates,^[^ ^19^ ^]^ would counteract the capacity of HS. Treating cells with 10 µm surfen, from day 0–2 or day 2–6 (**Figure** **5**A–D), significantly antagonized the marker genes expression of PS or PM, indicating the effect of HS interacting with signaling molecules at the stage of PS and PM formation (Figure 5B–E). To investigate the pathway of HS regulation at this stage, we screened the FGF, WNT, BMP, TGF‐β, and PDGF signaling pathways by treating cells with specific chemical inhibitors from day 0–2 or day 2–6 (Figure 5A–D; Table S4, Supporting Information). FGF signaling inhibitors remarkably decreased the expression of the marker genes *T*, *TBX6*, and *MSGN1* (Figure 5C–G).

Since FGFs were not added to the stimulating medium from day 0 of differentiation, we hypothesized that endogenous FGFs secreted by differentiated cells have a paracrine function to regulate FGF signaling. Transcriptome analysis by RNA‐seq indicated that paracrine or endocrine FGFs (FGF8, FGF17, and FGF19) were remarkably increased on day 2 of differentiation (Figure 5H), indicating the role of paracrine or endocrine FGFs in hiPSCs differentiation to PS and PM. To investigate the effects of p421 downstream of FGFR, we treated hiPSCs with a PI3K inhibitor (PIK90), ERK inhibitor (PD0325901), or PLCγ inhibitor (U73122) during PS or PM formation. ERK phosphorylation inhibitor remarkably decreased the marker gene expression in the PS (*T*) or PM (*TBX6*, *MSGN1*) (Figure 5I–K). In agreement with this, p421 was seen to enhance ERK1/2 phosphorylation when compared to LM421‐E8 during the paraxial mesoderm differentiation of hiPSCs (Figure 5L), indicating that HS regulating FGFR through its downstream ERK pathway.

### 2.5 P421 Initiates hiPSCs Differentiation via HS Regulating Exogenous bFGF in Pre‐Culture Stage

**Figure 6:**
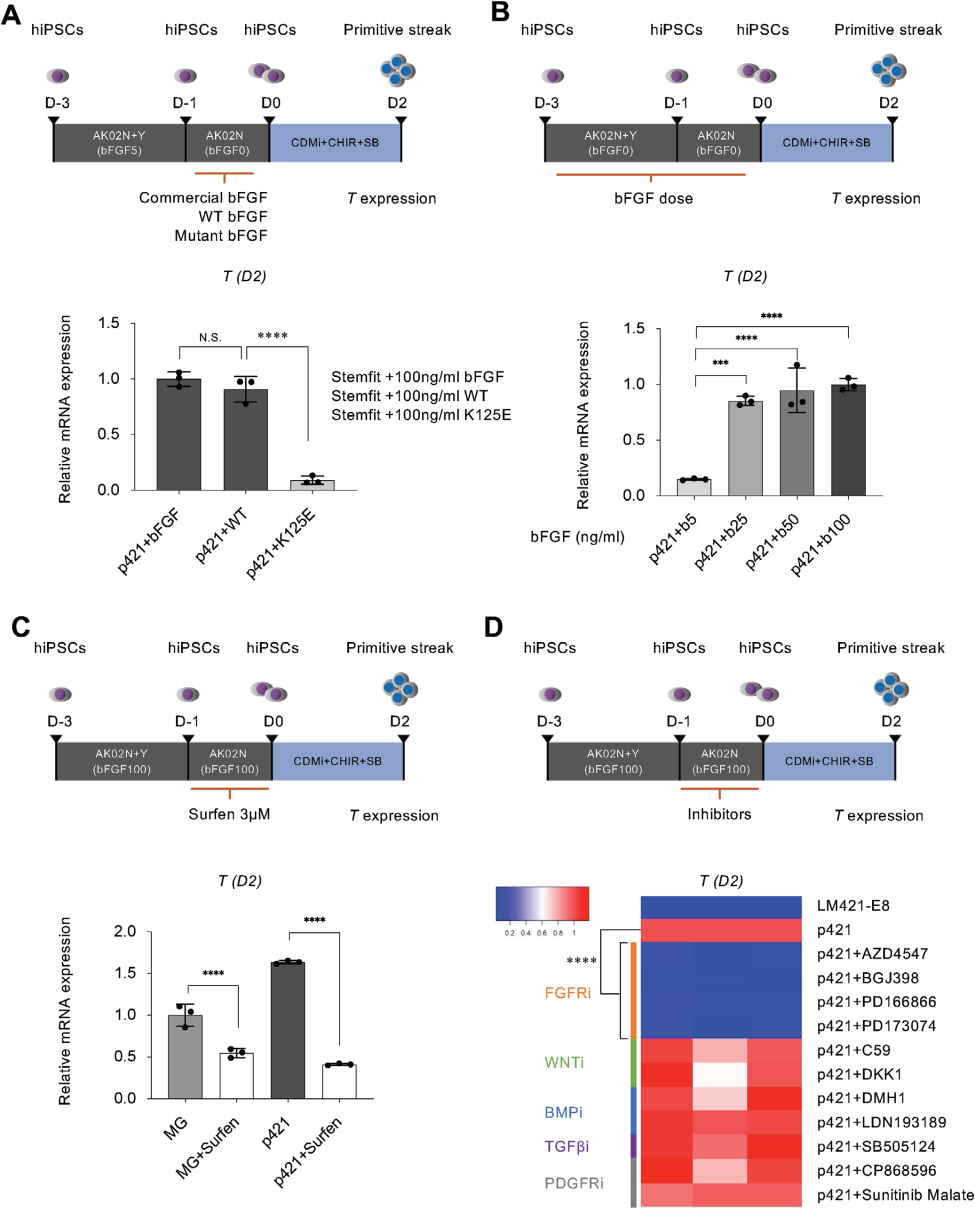
HS regulating bFGF triggers the hiPSCs differentiation in the pre‐culture stage. a) hiPSCs cultured on p421 were incubated in AK02N+Y with reduced bFGF (5 ng mL^−1^) for 2 days, then treated cells with commercial bFGF, recombinant WT bFGF, and K125E mutant bFGF for 1 day before stimulating differentiation. qRT‐PCR analysis comparing the expression level of *T* on day 2 after treatment with WT or mutant bFGFs. Error bars, mean ± SD, n = 3. b) hiPSCs cultured on p421 were incubated in AK02N without bFGF and treated with different doses of bFGF for 3 days before stimulating differentiation. A Dose‐dependent effect of bFGF (5, 25, 50, and 100 ng mL^−1^) from day ‐3–0 was detected by qRT‐PCR analysis comparing the expression level of *T* on day 2. c) Three micromolar surfen was added to StemFit (AK02N) containing 100 ng mL^−1^ bFGF for 1 day before stimulating differentiation. qRT‐PCR analysis comparing the expression level of *T* on day 2 in MG, LM421‐E8, or p421 treated with surfen. Error bars, mean ± SD, n = 3. d) Before stimulating differentiation, cells cultured on p421 were incubated with inhibitors for 1 day. FGF inhibitors: AZD4547 (1 µM), BGJ398 (1 µm), PD166866 (1 µm), and PD173074 (1 µm); Wnt inhibitors: Wnt‐C59 (1 µm) and DKK1 (1 µm); BMP inhibitors: DMH1 (1 µm) and LDN193189 (1 µm); TGFβ inhibitor: SB505124 (1 µm); PDGF inhibitors: CP868596 (1 µm) and Sunitinib Malate (1 µm). qRT‐PCR analysis comparing the expression level of *T* on day 2 after treatment with inhibitors. Error bars, mean ± SD, n = 3. *P*‐values were obtained using a one‐way ANOVA with Tukey's multiple comparisons test. ^*^ *P* < 0.05, ^**^ *P* < 0.01, ^***^ *P* < 0.001, ^****^ *P* < 0.0001. e) hiPSCs were transfected with GAPDH, HOXA1, or HOXB3 siRNA. After 2 days of transfection, the hiPSCs were seeded on p421‐coated dishes. The mRNA expression of *T* was analyzed by qRT‐PCR on day 2 of differentiation. *P*‐values were obtained using a student's t‐test. ^**^ *P* < 0.01, ^***^ *P* < 0.001, ^****^ *P* < 0.0001.

HS could interact not only with endogenous FGFs but also with exogenous FGFs.^[^ ^20^ ^]^ In this differentiation protocol, hiPSCs were cultured on p421 with AK02N (Stemfit) containing a high dose of exogenous bFGF (100 ng mL^−1^) in the pre‐culture stage (day ‐3–0). Because endogenous FGFs showed low expression and no difference between LM421‐E8 and p421 on day 0 (Figure S4A, Supporting Information), endogenous FGFs may not be the major determinant of the enhanced differentiation‐promoting activity of p421. So, we ask if HS mediates exogenous bFGF interactions with FGFR, thereby initiating hiPSCs differentiation. To confirm this hypothesis, we utilized mutated bFGFs, which contain an HS binding mutation (K125E), in the hiPSC maintenance medium. Mutated bFGF significantly decreased *T* expression on day 2 (**Figure** **6**A). When cells were treated with a low dose of bFGF from days ‐3–0, *T* expression decreased in a dose‐dependent manner (Figure 6B). Furthermore, treatment with surfen for 1 day before differentiation markedly blocked differentiation (Figure 6C). We also screened the FGF, WNT, BMP, TGFβ, and PDGF signaling pathways by treating cells with specific chemical inhibitors from the day ‐1–0. FGF signaling inhibitors also markedly decreased *T* expression on day 2 (Figure 6D). These results indicated that HS mediates exogenous bFGF to stimulate the FGF signaling pathway, thereby initiating hiPSCs to tend to differentiation at day 0.

Transcriptome analysis showed not much difference between LM421‐E8 and p421 on day 0; however, we did detect a collinear trend in the expression of *HOX* gene clusters, beginning with *HOXA1*, *HOXB3*, *HOXB1*, and *HOXB8* on day 0 (Figure S4B, Supporting Information) and culminating with *HOXA5*, *HOXB2*, *HOXB3*, *HOXB4*, *HOXB6*, *HOXB7*, *HOXB8*, *HOXB9*, and *HOXC4* on day 2 (Figure S3A, Supporting Information). *GAPDH* mRNA silencing experiment showed *GAPDH* expression was reduced on day 0 and recovered on day 2 (Figure S4D, Supporting Information), indicating the silencing effect of siRNA could be achieved for 5 days at 20 nm in hiPSCs. Silencing of *HOXA1* or *HOXB3* in the pre‐culture 5 days significantly antagonized the PS marker genes (*HOXA1*, *HOXB3*, and *T*) expression on day 2 (Figure S4E–H, Supporting Information). These observations suggest that the initial transcriptional availability of *HOX* genes depends on the HS mediating FGF pathway activity, which is activated in a temporal collinear fashion in undifferentiated hiPSCs to the PS differentiation.

### 2.6 The C‐Terminal Region‐Conjugated HS in p421 has a Significantly Higher Ability to Initiate and Stimulate hiPSC Differentiation

**Figure 7:**
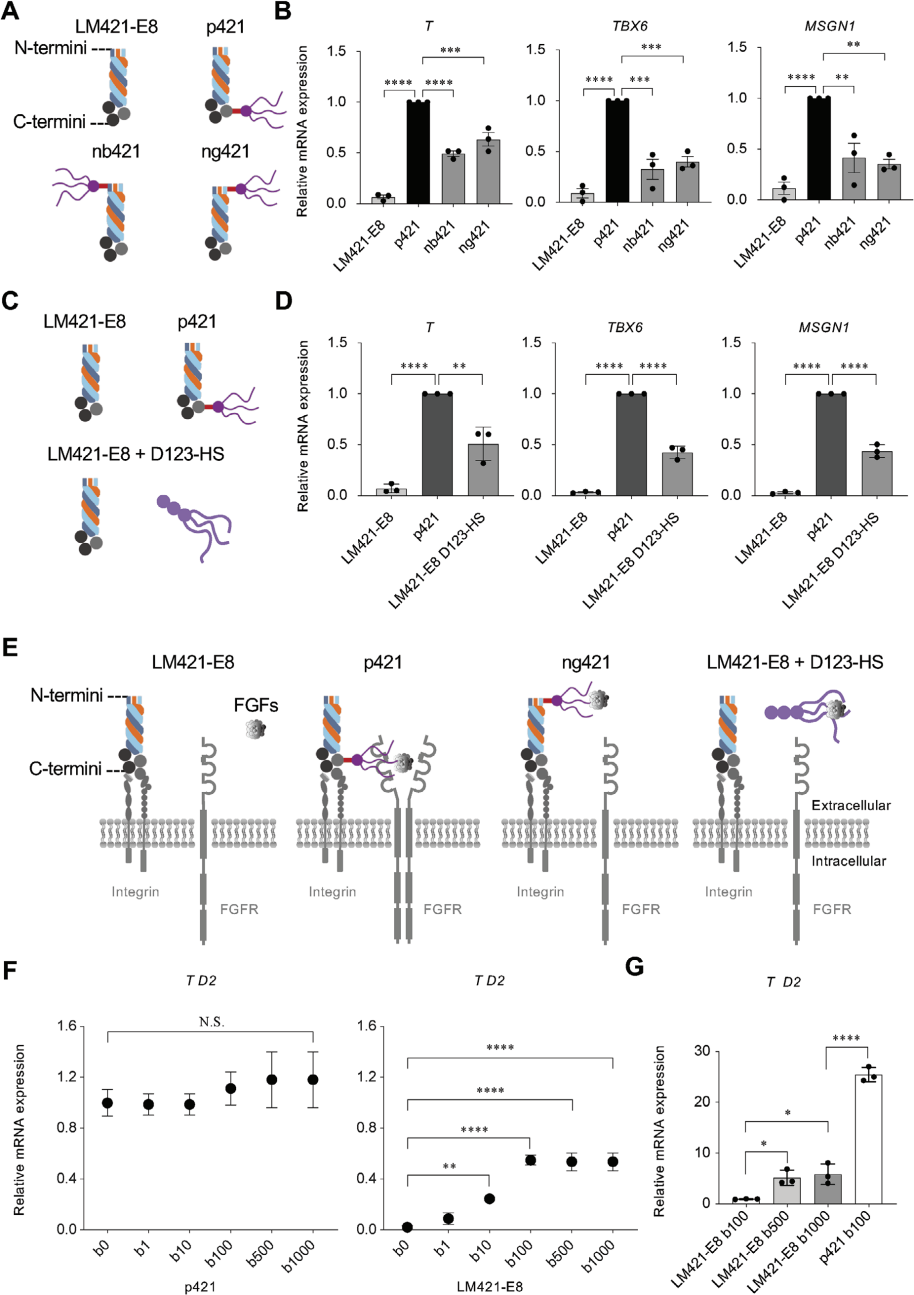
Effects of N‐terminal HS and D123‐HS in hiPSCs differentiation. a) Schematic illustration of the structure of LM421‐E8, p421, nb421 (HS conjugated to the N‐terminus of β chain in LM421‐E8), and ng421 (HS conjugated to the N‐terminus of γ chain in LM421‐E8). b) Gene expression of *T* (day 2), *TBX6*, and *MSGN1* (day 4) of cells cultured on LM421‐E8, p421, nb421, or ng421. Error bars, mean ± SD, n = 3. *P*‐values were obtained using a one‐way ANOVA with Tukey's multiple comparisons test. ^**^ *P* < 0.01, ^***^ *P* < 0.001, ^****^ *P* < 0.0001. c) Schematic illustration of the culture dishes coated with LM421‐E8, p421, and LM421‐E8 + D123‐HS (coated with LM421‐E8 and D123‐HS). d) Gene expression of *T* (day 2), *TBX6*, and *MSGN1* (day 4) of cells cultured on LM421‐E8, p421, and LM421‐E8 + D123‐HS. Error bars, mean ± SD, n = 3. *P*‐values were obtained using a one‐way ANOVA with Tukey's multiple comparisons test. ^**^ *P* < 0.01, ^****^ *P* < 0.0001. e) Schematic illustration showing the proximity of the LM‐E8, integrin, FGFs, and FGFRs. C‐terminal conjugated D1‐HS in LM421‐E8s brings the FGFs near to the FGFRs on the cell surface, facilitates a complex of FGFs‐FGFR‐HS, mediates the dimerization of FGFRs, then stimulates the FGFR signaling pathway. The N‐terminal conjugated D1‐HS or the separated coating D123‐HS brings the FGFs far to the cell surface, which could not fully stimulate the FGFR signaling pathway. f,g) bFGF dose and *T* gene expression titration curves, bFGF dose (b ng mL−1). f) hiPSCs were treated with varying levels of bFGF from days 0–2. g) hiPSCs were treated with varying levels of bFGF from day ‐3–0. f,g) qRT‐PCR analysis indicating the expression level of *T*. Error bars, mean ± SD, n = 3. *P*‐values were obtained using a one‐way ANOVA with Tukey's multiple comparisons test. ^*^ *P* < 0.05, ^**^ *P* < 0.01, ^****^ *P* < 0.0001; N.S.: not significant.

Heparin/HS can enhance the binding of bFGF to FGFR and dimerization of FGFR.^[^ ^21^, ^22^, ^23^, ^24^ ^]^ The C‐terminal region of LM‐E8 fragments comprises the binding site for integrins ^[^ ^13^, ^25^ ^]^ and is positioned in close proximity to the cell surface (**Figure** **7**E). To examine whether the C‐terminally conjugated HS has stronger effects in stimulating FGFR in hiPSC differentiation, we compared p421 with nb421 or ng421, in which HS was conjugated to the N‐terminal region of the β‐chain or γ‐chain (Figure 7A). Although nb421 and ng421 increased *T, TBX6*, and *MSGN1* expression, p421 showed much stronger effects on PS and PM differentiation from hiPSCs (Figure 7B–E). Similarly, compared to the dish coated with the mixture of LM421‐E8 and D123‐HS (Perlecan domain 1, 2, 3 with HS), p421 showed significantly higher effects in stimulating hiPSC differentiation (Figure 7C,D). The protein levels of T and TBX6 were similar to the gene expression levels (Figure S8A–D, Supporting Information). We also added D1‐HS (perlecan D1 domain with heparin sulfate chains) or heparin to the culture medium of hiPSCs cultured in LM421‐E8. D1‐HS and heparin increased *T, TBX6*, and *MSGN1* expression, but they could not get the level of p421 (Figure S8E, Supporting Information). These results suggest that conjugation of D1‐HS to the C‐terminus of LM421‐E8s brings the HS‐bound FGFs in close proximity to the cell surface, thereby facilitating a complex of FGFs‐FGFR‐HS on the cell surface that stimulates the FGFR signaling pathway by FGFR dimerization (Figure 7E).

Next, we examined the effect of high‐dose bFGF on the initiation and early stages of differentiation. Treatment with bFGF from day 0–2 dose‐dependently increased *T* expression in LM421‐E8 cells, with a maximum effect reached at 100 ng mL^−1^ (Figure 7F); there were no significant differences among the groups in p421 (Figure 7F). Very high doses of bFGF (1 mg mL^−1^) increased *T* expression in LM421‐E8 cells; however, it did not reach the level of the p421 group, even in p421 cells without bFGF treatment (Figure 7F). Next, we examined whether incubation of undifferentiated hiPSCs with high‐dose bFGF from day ‐3–0 could rescue subsequent differentiation. Treatment with high‐dose bFGF in LM421‐E8 cells at the undifferentiated stage increased the expression of *HOXA1* (day 0), *HOXB1* (day 0), and *HOXB3* (day 0) (Figure S5A–C, Supporting Information), however, it could not fully rescue the differentiation of hiPSCs cultured on p421 (Figure 7G; Figure S5D–I, Supporting Information). These results indicate that the C‐terminally conjugated HS in p421 has a significantly higher ability to initiate and stimulate hiPSCs differentiation, which could not be comparable by treating with high dose bFGF or with Heparin/HS, suggesting p421 is a unique structure for regulating hiPSCs differentiation.

### 2.7 Differentiation Reproducibility in p421 with hiPSCs from Patient‐Derived Cell Origins

**Figure 8:**
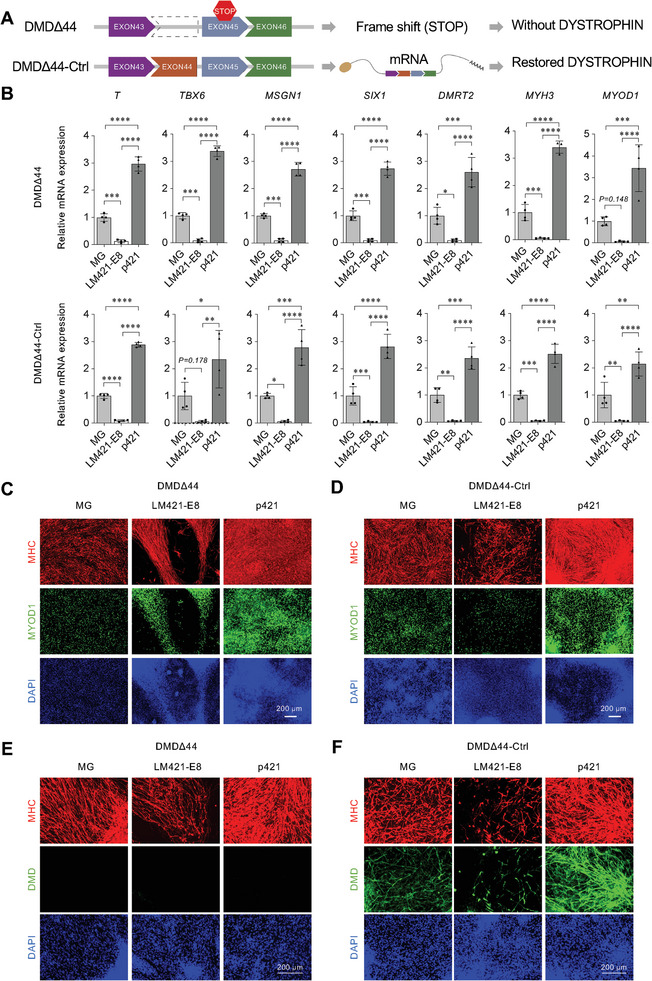
Effects of p421 in myogenic differentiation of DMD patient‐derived iPSCs. a) Schematic illustration of the DMD patient‐derived hiPSC line (DMDΔ44) and the DMD‐corrected isogenic control cell line (DMDΔ44‐Ctrl). b) qRT‐PCR analysis comparing the expression level of *T* (day 2), *TBX6, MSGN1* (day 4), *SIX1, DMRT2* (day 14), *MYH3*, and *MYOD1* (day 38) on MG, LM‐E8s, or p421. Error bars, mean ± SD, n = 3. *P*‐values were obtained using a one‐way ANOVA with Tukey's multiple comparisons test. ^*^ *P* < 0.05, ^**^ *P* < 0.01, ^***^ *P* < 0.001, ^****^ *P* < 0.0001. c,d) MHC, MYOD1, and DAPI staining of DMD‐ and CKI‐derived myocytes (day 38) in MG, LM421‐E8, and p421. e,f) MHC, DMD, and DAPI staining of DMD‐ and DMDΔ44‐Ctrl ‐derived myocytes (day 38) in MG, LM421‐E8, and p421.

Current differentiation protocols lack consistency among hiPSC lines. To address this limitation, we generated multiple hiPSC lines from two types of muscular dystrophy: Duchene muscular dystrophy (DMD) and Miyoshi myopathy (MM). DMD is inherited in an X‐linked pattern because mutations in the *DYSTROPHIN* gene (*DMD)* are located on the short arm of the X chromosome. The DMD line was established from a patient with lacking EXON44 in the *DMD* gene (DMDΔ44), and the CRISPR‐Cas9 knock‐in isogenic control line (DMDΔ44‐Ctrl) was inserted with EXON44 via genome editing (**Figure** **8**A).^[^ ^4^, ^26^, ^27^ ^]^ MM patient‐derived hiPSCs showed heterozygous mutations in each allele of the *DYSFERLIN* gene (Figure S6A, Supporting Information), causing a lack of the DYSFERLIN (DYSF) protein and progressive muscle disorders. The control hiPSC line of MM was established from the healthy younger sister of the MM patient, who had a missense mutation in one allele (Figure S6A, Supporting Information).^[^ ^28^, ^29^ ^]^ Each hiPSC line was cultured in MG‐, LM421‐E8‐, or p421‐coated dishes. As expected, p421 produced significantly higher efficiencies in marker gene expression at each stage and myocyte formation (Figure 8B–D; Figure S6B–D, Supporting Information). We also assessed the expression of DYSTROPHIN in both DMDΔ44 and DMDΔ44‐Ctrl myocytes and DYSF expression in both MM and control myocytes. Immunocytochemistry confirmed that the DYSTROPHIN protein was colocalized with MHC in DMDΔ44‐Ctrl myocytes but not in DMDΔ44‐myocytes (Figure 8E,F). Immunostaining of DYSF in MM cells was very faint, whereas DYSF was merged with MHC in control myocytes (Figure S6E,F, Supporting Information). p421 showed good myogenic differentiation in each of these hiPSC lines, indicating that using p421 we could establish a universal and robust differentiation protocol for myogenic differentiation from multiple hiPSC lines.

### 2.8 Differentiation Reproducibility in p421 with hiPSCs Cultured in mTeSR1 or E8

In our cell culture system, hiPSCs were cultured in StemFit AK02N medium. However, mTeSR1 and E8 media are also frequently used by other research groups. To investigate whether p421 enhances muscle differentiation in hiPSCs cultured in other stem cell media, we tested hiPSCs cultured in mTeSR1 or E8. p421 significantly increased the expression of marker genes at each PS stage (*T*), PM (*TBX6* and *MSGN1*), DM (*SIX1* and *DMRT2*), and myocytes (*MYH3* and *MYOD1*) (Figure S9A,B, Supporting Information), indicating that p421 can be used in the myogenic differentiation protocol for hiPSC lines cultured in mTeSR1 or E8.

## 3 Discussion

Here, we established a new system for hiPSC differentiation on p421, a recombinant form of the D1‐HS of perlecan conjugated to the C‐terminus of LM421‐E8.

Laminin‐511 has been used as an extracellular matrix for hiPSCs culture,^[^ ^10^ ^]^ and LM‐E8s, which serve as a functionally minimal form of laminin, have been established as xeno‐free substrates for maintaining undifferentiated hiPSCs.^[^ ^13^, ^14^ ^]^ However, without the contribution of HSPGs, each type of LM‐E8s shows significantly lower efficient myogenic differentiation compared with MG, indicating the role of heparan sulfate proteoglycans in myogenic differentiation in vitro. To solve this problem for clinical application, we conjugated the D1 of perlecan with HS to the LM‐E8 fragment, generating NGLFs, which remarkably increased the efficiency of hiPSCs toward paraxial mesoderm and muscle lineage.

LM511‐E8 or Matrigel is the most used commercially available substrate for iPSC feeder‐free maintenance or iPSC differentiation.^[^ ^8^, ^14^ ^]^ In this study, we have compared p421 with MG and LM511‐E8 and found that p421 showed high efficacy for skeletal muscle differentiation. Collagen is the most abundant extracellular matrix protein in vertebrates and is widely used for skeletal muscle cultures in vitro. However, hiPSCs regularly adhere to LM421‐E8/LM511‐E8‐coated substrates and proliferate in an integrin α6β1‐dependent manner, but they cannot proliferate on collagen‐coated substrates.^[^ ^30^ ^]^ Similar to collagen, Laminin‐211 is also highly expressed in skeletal muscle tissue. However, Laminin‐211 also exhibits very low affinity to integrin α6β1, which is the major integrin expressed in hiPSCs, and therefore does not support the adhesion of hiPSCs onto culture plates.^[^ ^31^ ^]^ Thus, for hiPSCs to induce mesoderm differentiation and myogenesis following human embryonic development, p421 is better than other commercially available substrates.

HS biosynthetic enzyme expression is developmentally orchestrated and is necessary for lineage commitment and cell fate decisions in embryonic stem cells.^[^ ^32^ ^]^ HS ablation in mouse embryonic stem cells (ESCs) failed to differentiate into PS and mesodermal progenitor cells.^[^ ^33^ ^]^ In agreement with the in vivo observations, our study shows that HS depletion by heparinase treatment disrupts PS differentiation, indicating the beneficial effect of HS in the paraxial mesoderm differentiation of hiPSCs.

Several extrinsic signaling pathways, including FGF, BMP, PDGF, TGFβ, and Wnt, promote mesoderm differentiation from ESCs.^[^ ^33^, ^34^, ^35^, ^36^ ^]^ Interestingly, by screening the signaling pathways, we observed that HS promoted FGF but not BMP, PDGF, TGFβ, or Wnt signaling in this culture condition. It is well known that HS functions as a co‐receptor to induce FGFR dimerization and facilitate FGF signaling,^[^ ^21^, ^37^, ^38^, ^39^, ^40^ ^]^ and activation of FGF signaling is essential for early vertebrate embryogenesis.^[^ ^16^, ^41^, ^42^ ^]^ We observed that incubation with a high dose of bFGF in the undifferentiated or early differentiation stages partially rescued PS and PM differentiation on LM‐E8‐coated dish, demonstrating the role of HS in regulating the FGF signaling pathway in early vertebrate embryogenesis in vitro.

Heparin/HS can enhance the binding of bFGF to FGFR and stimulate the FGF signaling in vitro.^[^ ^21^, ^22^, ^23^, ^24^ ^]^ Coating with LM421‐E8 having N‐terminally added HS or with a mixture of LM421‐E8 and D123‐HS could not show the same effects as p421, underscoring the importance of the unique structure of NGLFs. The C‐terminal region of LM‐E8 fragments, comprising the laminin globular 1–3 domains of the α chain and the C‐terminal tail of β and γ chain, are prerequisites for the integrin binding activities of laminins.^[^ ^25^ ^]^ Among the NGLFs, p411, p421, p511, and p521 showed high efficiency in myogenic differentiation, indicating the role of specific laminin‐integrin interactions in the early differentiation of hiPSCs. As hiPSCs abundantly express α6β1 integrins and integrin α6 or β1 neutralization antibody blocked hiPSC attachment (data not shown),^[^ ^10^ ^]^ we surmise that hiPSCs can attach quickly and migrate efficiently on LM411‐E8‐, LM421‐E8‐, LM511‐E8‐, or LM521‐E8‐coated dish. Integrins also engage in reciprocal crosstalk with growth factor receptors.^[^ ^43^, ^44^ ^]^ Growth factor receptors can be clustered and dimerized through a ligand‐dependent or ‐independent pathway when integrins and their receptors are in relative proximity, inducing integrins to mediate the phosphorylation of growth factor receptors and activate ERK signaling.^[^ ^41^, ^43^, ^44^ ^]^ In line with this mechanism, conjugation of D1‐HS to the C‐terminus of LM‐E8s brings the FGFs bound to the HS chains of the D1 domain close to the integrin binding site of laminin E8 fragments, thereby could facilitate co‐stimulation of the signaling pathways downstream of FGFRs and integrins, leading to the promotion of myogenic differentiation of hiPSCs. We hypothesized that the unique structure of p421 juxtaposes the laminin‐bound integrin α6β1 to the ligand‐bound FGFR and facilitates a tetrameric complex of HS‐FGFR‐laminin‐integrin on the cell surface, therefore, activating the phosphorylation of FGFR and its downstream ERK pathway.

FGFs are potent regulators of the proliferation and differentiation of hiPSCs.^[^ ^45^ ^]^ In the LM511‐E8 feeder‐free culture system, high concentration bFGF (100 ng mL^−1^) supports hiPSCs self‐renewal and staying in primed state.^[^ ^45^, ^46^ ^]^ Before stimulating differentiation, hiPSCs were pre‐cultured in AK02N with exogenous 100 ng mL^−1^ bFGF on p421 for 3 days. The binding of bFGF in AK02N to HS of p421 significantly activates the FGF signaling prior to differentiation and, therefore triggers the onset of HOX gene activation in hiPSCs. In agree with these results, in vivo studies showed Hox gene was activated in the epiblast at the beginning of gastrulation in early chicken embryo development.^[^ ^47^, ^48^ ^]^ FGF signaling shows high activity in PS and PM in embryonic development in vivo.^[^ ^42^, ^49^, ^50^ ^]^ In this in vitro differentiation system, bFGF was removed from the differentiation medium from day 0, however, paracrine or endocrine FGFs were highly expressed in this stage. Endogenous FGFs bind to HS of NGLFs stimulating FGF singling pathway activation.

Hox genes are activated in a temporal collinear fashion in the epiblast lateral to the PS in chick embryogenesis, controlling the timing of epiblast cell ingression into the PS and PM.^[^ ^8^, ^48^, ^51^, ^52^, ^53^, ^54^, ^55^ ^]^ In agreement with the in vivo study, we detected that the collinear activation of 3′ HOX genes (*HOXA1*, *HOXB1*, and *HOXB3*), and culminated with 5′ HOX genes (*HOXA5, HOXB5, HOXB5, HOXB6, HOXB7, HOXB8, HOXB9, HOXC4, HOXC6*). The differentiation of hiPSCs into a PS fate on p421 recapitulates a developmental sequence similar to embryogenesis in vivo, leading to the production of PS and PM cells. Interestingly, the exogenous bFGF in hiPSCs maintenance medium activates the onset of HOX gene expression, therefore initiating hiPSC differentiation.

FGF signaling plays a pivotal role in the regulation of cell movement and lineage induction during gastrulation in vivo. In this study, fully activating FGF signaling in NGLFs induced hiPSC differentiation into the paraxial mesoderm, whereas reduced FGF in LM‐E8 induced neurogenesis in hiPSCs. These data suggest that LM‐E8 may be more suitable for the neural differentiation of hiPSCs. However, controlling the level of FGF signaling for neural differentiation of hiPSCs requires further investigation.

Muscular dystrophies are a group of rare disorders that cause progressive muscle weakness and degeneration. hiPSCs are an attractive cell source for disease modeling of muscular dystrophies. Recently, we induced hiPSCs to generate myotubes with mature myotube characteristics, such as sarcomere, triad structures, and calcium homeostasis, which is a great model for muscular diseases and drug screening.^[^ ^56^ ^]^ However, the variations among hiPSCs, such as the types of original cells or the genetic backgrounds of the donor cells, induce diversity in their differentiation propensity toward muscle lineages.^[^ ^3^, ^4^, ^5^, ^6^, ^57^, ^58^, ^59^, ^60^ ^]^ In the current study, we examined the myogenic differentiation of multiple muscular dystrophy patients derived hiPSC clones. Various hiPSCs can be effectively and stably differentiated into myocytes and MuSCs, showing great potential for disease modeling and clinical application.^[^ ^3^, ^4^, ^5^, ^6^, ^57^, ^58^, ^59^ ^]^

In conclusion, using the recombinant NGLFs, we established a more efficient protocol for generating myocytes and MuSCs in different types of hiPSCs. Moreover, we revealed that HS of NGLFs binding with exogenous bFGF and endogenous FGFs potentiates myogenic differentiation from hiPSCs by regulating the FGF signaling pathway. HS of NGLFs regulates hiPSCs differentiation in the stage prior to the PM formation, thereby generating purer PM‐like cells, which is beneficial for PM‐derived lineage differentiation. Taking advantage of NGLFs, we established a highly efficient and xeno‐free matrix‐based protocol for muscle differentiation of hiPSCs, providing an attractive cell source for skeletal muscle disease modeling and clinical cell therapies.

## 4 Experimental Section

### Ethical Approval

Ethical approval for this study was granted by the Ethics Committee on Human Stem Cell Research, Institute for Frontier Medical Sciences, Kyoto University, and Kyoto University Hospital.

### Human iPSC Lines and Maintenance Culture

HiPSC clones 201B7, 414C2, DMDΔ44, DMDΔ44‐Ctrl, MM, and MM‐Control^[^ ^4^, ^26^, ^29^ ^]^ were cultured and maintained in feeder‐free culture on iMatrix‐511 (Nippi) in StemFit AK02N medium (Ajinomoto) as previously described.^[^ ^6^ ^]^ The 201B7 and 414C2 cell lines were used as parental cell lines to generate Myf5‐tdTomato reporter lines.

### Plate Coating with NGLFs

Into the wells of a 6‐well plate, 1.5 mL of phosphate‐buffered saline (PBS (−)) was dispensed. Next, LM‐E8s or NGLFs were added to a final concentration of 6.6 pmol cm^−2,^ and the solutions were mixed immediately. The plates were incubated at 37 °C for at least 1 h or 4 °C overnight. The LM‐E8s or NGLFs suspensions were aspirated and the plate was immediately used for cell seeding.

### Generation of NGLFs

Expression vectors for recombinant human LMa1‐E8, a2‐E8, a3‐E8, a4‐E8, a5‐E8, b1‐E8, b2‐E8, b3‐E8, g1‐E8, g2‐E8, and a5‐E8 C‐terminally conjugated with the D1 domain of perlecan (cPa5‐E8) were prepared as described previously.^[^ ^12^, ^13^, ^61^, ^62^, ^63^, ^64^, ^65^ ^]^ Full‐length human perlecan (fhPerlecan) was prepared as previously described.^[^ ^64^ ^]^ The 6×His, HA, and FLAG tags were added to a‐E8, b‐E8, and g‐E8, respectively, at their N‐termini. Expression vectors for cPa1/2/3/4‐E8, comprising LMα1/2/3/4‐E8 and the D1 domain connected through a linker segment derived from LM‐α1 (Asp2684‐Pro2698) were also generated as previously described.^[^ ^61^ ^]^ Briefly, cDNA fragments encoding Pa‐E8 were amplified by extension PCR using the expression vectors for LMa1/2/3/4‐E8 and cPa5‐E8 as templates. The resultant cDNA fragments were assembled into NheI/NotI‐cleaved pcDNA3.4 (Thermo Fisher Scientific) containing the multiple cloning site (mcs) derived from pSecTag2A (pcDNA3.4‐mcs).^[^ ^25^ ^]^ To construct expression vectors for LMb1‐E8 and g1‐E8 with an N‐terminally fused D1/[GSGGG]_4_ linker and a FLAG tag (nPb1‐E8 and nPg1‐E8), cDNA fragments encoding nPb1‐E8 and nPg1‐E8 were amplified by extension PCR using expression vectors for LMb1‐E8, LMg1‐E8, and fhPerlecan as templates. The resultant cDNAs were cleaved using NheI/NotI and ligated into NheI/NotI‐cleaved pcDNA3.4‐mcs. An expression vector for LMb2‐E8 with an N‐terminally fused D1 and a FLAG tag (nPb2‐E8) was prepared by extension PCR using expression vectors for nPb1‐E8 and LMb2‐E8 as templates. The resultant cDNA fragment was assembled into the NheI/NotI‐cleaved pcDNA3.4. For an expression vector for HA‐tagged LMg1‐E8, a cDNA encoding the Igk leader sequence/HA tag/LMg1‐E8 was amplified by extension PCR using an expression vector for LMg1‐E8 as a template, and the resultant cDNA fragment was then assembled into a HindIII/NotI‐cleaved pcDNA3.4 expression vector for LMg1‐E8. An expression vector for recombinant D123 of human perlecan (D123‐HS) was constructed by inserting a cDNA encoding D123‐HS (V22‐P1676) with a C‐terminal 6×His tag, which was amplified by PCR using an expression vector for fhPerlecan as a template, into NheI/NotI‐cleaved pSecTag2A. The DNA sequences of all expression vectors were verified using an ABI PRISM 3130xl Genetic Analyzer (Thermo Fisher Scientific). The cDNA sequences of the p421 component are presented in Figure S11 (Supporting Information).

All recombinant proteins were produced using the FreeStyle 293 Expression System (Thermo Fisher Scientific) and purified from conditioned media, as reported previously.^[^ ^12^ ^]^ Conventional laminins (LM111‐E8, LM211‐E8, LM332‐E8, LM411‐E8, LM421‐E8, LM511‐E8, and LM521‐E8) were purified by sequential chromatography using Ni‐NTA‐agarose (Qiagen) and anti‐FLAG M2‐agarose (Sigma) columns. Next‐generation laminins (p111, p211, p332, p411, p421, p511, p521), nb421, ng421, and D123‐HS were purified in the same manner, except that salt washing using TBS (−) containing 1 m NaCl was included in the Ni‐NTA‐agarose purification step. D123‐HS was purified using single Ni‐NTA‐agarose chromatography. All purified proteins were dialyzed against PBS (−) (pH 7.4). Protein concentrations were determined using a BCA protein assay kit (Thermo Fisher Scientific), with bovine serum albumin (BSA) as a standard.

### Myogenic Differentiation

HiPSCs were dissociated with Accutase (Nacalai) and plated as single cells on MG‐coated, Laminin‐E8‐coated, or NGLF‐coated 6‐well plates (10 000 cells per well) in Stemfit (AK02N, Ajinomoto) supplemented with a ROCK inhibitor (10 µm, Y‐27632, Sigma) for 2 days. The medium was replaced with fresh Stemfit, and the cells were kept for one day. Next, the medium was changed to a differentiation medium, which contained CDMi supplemented with CHIR99021 (CHIR, Axon MedChem, Tocris) and SB431542 (SB, Sigma). CDMi contained IMDM (+) *L*‐glutamine (+) 25 mm HEPES (Invitrogen, 12440053) and F12 (1X) Nutrient Mixture (Ham) (+) *L*‐Glutamine (Invitrogen, 11765054) at a ratio 1:1 supplemented with 1% (w/v) BSA (Sigma), 1% penicillin‐streptomycin mixed solution (Nacalai), 1% CD Lipid Concentrate (Invitrogen), 1% Insulin‐Transferrin‐Selenium (Invitrogen), and 450 µm 1‐Thioglycerol (Sigma). After 7 days of differentiation, the cells were dissociated with Accutase (Nacalai) and plated as single cells on MG‐coated, Laminin‐E8‐coated, or NGL‐coated 6‐well plates (400 000 cells per well) in CDM supplemented with CHIR, SB, and ROCK inhibitor (10 µm Y‐27632, Sigma). On differentiation day 14, cells were dissociated with Accutase (Nacalai) and plated as single‐cell MG‐coated, Laminin‐E8‐coated, or NGL‐coated 6‐well plates (400 000 cells per well) in CDM supplemented with Y‐27632. The medium was changed to serum‐free culture medium (SF‐O3; Sanko Junyaku) supplemented with 0.4% BSA, 0.1 mm 2‐mercaptoethanol (2‐ME), 10 ng mL^−1^ recombinant human IGF‐1 (PeproTech), 10 ng mL^−1^ recombinant human bFGF (Oriental Yeast Co., LTD, NIB 47079000), and 10 ng mL^−1^ recombinant human HGF (PeproTech). The medium was changed every two or three days from day 17–38. Finally, the medium was changed to DMEM (Invitrogen, 11960069) supplemented with 0.5% penicillin‐streptomycin (Nacalai, 26253–84), 2 mm *L*‐glutamine (Nacalai, 16948‐04), 0.1 mm 2‐ME, 2% horse serum (Sigma), 5 µm SB, and 10 ng mL^−1^ IGF‐1 (Peprotech) for 12 weeks. 10 000 cells were seeded in each well of a 6‐well plate for differentiation. On differentiation day 14, ≈5 000 000 cells were harvested from each well of the 6‐well‐plate. In each batch of experiments, 12 wells (400 000 cells per well) in 6‐well plates were cultured from day 14. After 12 weeks of culturing, ≈2 000 000 cells were harvested from each well. Thus, 24 000 000 cells were obtained after 12 weeks of culture.

### Cell Preparation for Fluorescence‐Activated Cell Sorting (FACS) Analysis

After 80 days of differentiation, the cells were treated with collagenase‐1 for 30–60 min at 37 °C and neutralized with DMEM +2% Horse serum. The cells were then centrifuged at 1000 rpm at 4 °C for 10 min. Cells before 6 weeks of differentiation did not undergo this collagenase treatment. After removing the supernatant, the cells were treated with Accutase at 37 °C for 5 min and neutralized with DMEM +2% Horse serum. The cells were then centrifuged at 1000 rpm at 4 °C for 10 min. After the removal of the supernatant, the cells were suspended in an HBSS buffer containing 1% Hoechst and filtered through a 40 µm mesh. The prepared cells were stored on ice before the FACS analysis.

### Flow Cytometry Analysis

Flow cytometry analysis was performed using Aria II (BD Biosciences) according to the manufacturer's protocol. Gating was determined for the MYF5‐tdTomato cell line using hiPSCs and an undifferentiated culture as the baseline control.

### RNA Extraction and Quantitative Real‐Time RT‐PCR (qRT‐PCR)

Total RNA was extracted using the ReliaPrep RNA Cell Miniprep System (Promega, Z6012), and cDNA was synthesized using the ReverTra Ace qPCR RT kit (TOYOBO, FSQ‐101). qRT‐PCR was carried out with the SYBR Green system (Applied Biosystems) and a One Step thermal cycler (Applied Biosystems) and was performed in triplicate for each sample. β‐ACTIN was used as an internal control. Primer sets used in this study are listed in Table S1 (Supporting Information).

### Immunocytochemistry

Differentiated cell samples were fixed with 2% paraformaldehyde (PFA; Nacalai Tesque) for 10 min at 4 °C in a culture dish. Sorted cells were seeded onto glass slides with Smear Gel (GenoStaff) and fixed with 2% PFA for 10 min at 4 °C. The cells were washed twice with PBS and blocked with Blocking One (Nacalai) for 30 min at 4 °C. The cells were stained with the appropriate primary antibodies diluted in 10% Blocking One in PBS for 16 h at 4 °C. After three washes with 0.2% Triton X‐100 (Sigma–Aldrich) in PBS (PBST), cells were stained with the appropriate secondary antibodies for 1 h at room temperature. DAPI, a nuclear stain (Sigma–Aldrich), was loaded at a 1:5000 dilution for 5 min. All of the antibodies used are listed in Table S2 (Supporting Information).

### Microarrays Analysis

Total RNA was isolated using a ReliaPrep RNA Miniprep System (Z6012). Sequencing libraries were constructed using the TruSeq Stranded mRNA Library Prep Kit (Illumina) and sequenced in the 100‐cycle single‐read mode of HiSeq2500. All sequenced reads were extracted in FASTQ format using the BCL2FASTQ Conversion Software 1.8.4 in the CASAVA 1.8.2 pipeline. FASTQ‐converted reads were mapped to hg19 reference genes using TopHat v2.0.8b and quantified using RPKMforgenes. Analyses were performed using R ver. 3.1.0. Heat maps of embryonic and fetal muscle progenitor marker expression were generated using Genespring GX 13 software.

### siRNA Transfection

For the knockdown experiments, 5 × 10^4^ cells were seeded in a single cell condition in one well of a 24‐well plate with AK02N+Y and without P/S. After overnight culturing, cells were transfected with the corresponding siRNAs (20 nm) using the Lipofectamine RNAiMAX reagent (Thermo Fisher Scientific). Twenty‐four hours after transfection, the medium was changed, and the cells were cultured for one more day and prepared for cell seeding for myogenic differentiation. siRNAs were purchased from Ambion (Life Technologies), and the siRNA sequences are provided in Table S3 (Supporting Information).

### Signaling Pathway Modulators

The major developmental signaling pathway modulators used in this study are listed in Table S4 (Supporting Information).

### Generation of Recombinant MBP‐bFGF Proteins

bFGF cDNA was cloned by PCR using human iPS cell (1383D2) cDNA as a template. The amplified fragment was cloned into pENTR1A (Thermo Fisher Scientific) using an In‐Fusion HD Cloning Kit (Takara). bFGF‐K125E mutant was generated using PCR‐based mutagenesis. bFGF‐WT or ‐K125E were then cloned into pMAL‐c5X (New England Biolabs) using an In‐Fusion HD Cloning Kit.

The primers pENTR1A‐bFGF‐FW and ‐RV were used to clone the bFGF gene into pENTR1A; bFGF‐K125E‐FW and ‐RV were used to insert mutations; and pMAL‐c5X‐bFGF‐FW and ‐RV were used for cloning of bFGF‐WT or K125E into pMAL‐c5X. The complete pMAL‐c5X‐bFGF sequence is shown in Figure S10 (Supporting Information). The primers used are listed in Supplementary Table S5 (Supporting Information).

The pMAL‐c5X‐bFGF plasmids were transformed into BL21(DE3) pLysS Competent Cells (Promega), and the cells were cultured in LB broth medium (Sigma) at 37 °C. When the OD_600_ value reached ≈0.8, isopropyl‐β‐D‐thiogalactopyranoside (IPTG, Nacalai) was added to the final concentration of 1 mm to induce the expression of the protein for 1 h. The cells were harvested by centrifugation, and the supernatant was removed. The pellet was resuspended in homogenization buffer (20 mm Tris/HCl (pH 7.6) (Nacalai Tesque), 1 mm ethylenediaminetetraacetic acid (EDTA), 1 mm DTT (Nacalai Tesque), 150 mm NaCl (Nacalai Tesque), 100 mm *D*‐(+)‐sucrose (Nacalai Tesque), and protease inhibitor cocktail (Nacalai Tesque)). The cells were then lysed by sonication. The homogenate was centrifuged at 4 °C, and the supernatant was applied to amylose resin (New England Biolabs). The resin was washed with wash buffer (20 mm Tris/HCl (pH 7.6), 1 mm EDTA, 1 mm DTT, and 150 mm NaCl) to remove impurities. To elute the recombinant bFGFs, elution buffer (20 mm Tris/HCl (pH 7.6), 1 mm EDTA, 1 mm DTT, 150 mm NaCl, and 20 mm maltose monohydrate) was added to the resin. The purified recombinant MBP‐bFGF proteins were filtered through 0.22 µm Millex‐GV Syringe Filter Units (Merck Millipore) and stored at −80 °C.

### HS in p421 Digestion

The dishes were coated with p421 overnight at 4 °C and washed three times with 0.1% BSA/TBS. The dishes were then incubated with a blocking buffer (1% BSA/TBS) at room temperature for 1 h. Next, the dishes were washed three times with 0.1% BSA/TBS and treated with 14.6 U µL^−1^ of heparinase from *Flavobacterium heparinum* (*F. heparinum*) (Amsbio, Code#:100703) in 2 mm CaCl_2_/TBS for 2 h at 37 °C. The dishes were washed three times with 0.1% BSA/TBS before use.

### Western Blotting Analysis

Cells were lysed in radio‐immunoprecipitation assay (RIPA) buffer (08714‐04, Nacalai Tesque) with protease inhibitor cocktail (25955‐11, Nacalai Tesque) by thorough sonication (UCD‐250, Bioruptor) at 4 °C. 20 µg of protein from cells were mixed with reducing agent (NP0004, Invitrogen, Carlsbad, CA, USA) and loaded onto 4%–12% Bolt Bis‐Tris Plus Gels (NM04122BOX, Thermo Fisher Scientific). The fractionated proteins were transferred to a PVDF membrane using the iBlot system (IB 401002 iBlot Transfer Stack, Thermo Fisher Scientific). The membrane was blocked with Blocking One (Nacalai Tesque) and incubated with primary antibody, which was diluted with Can Get Signal Solution 1 (NKB‐201, TOYOBO) at 4 °C overnight. After three washes with TBS with 0.05% TWEEN 20 (P7949, Sigma–Aldrich), the membrane was incubated with secondary antibody, which was diluted with Can Get Signal Solution 2 (NKB‐101, TOYOBO), for 1 h at room temperature. Detection was carried out with SuperSignal West Femto Substrate (34094, Thermo Fisher Scientific). Visualization and semi‐quantification of the images were performed using the Image Quant LAS4000 imaging system (GE healthcare, Chicago, IL, USA).

### Statistical Analysis

At least three independent experiments were performed. All data were included in the individual data plots. Two‐group comparisons were performed using the unpaired t‐test. Comparisons of more than two groups were performed using one‐way ANOVA, followed by Tukey's multiple comparison. Differences between groups were considered significant at a *P* value of < 0.05. The statistical analyses were performed using GraphPad Prism 8.0 (GraphPad Software Inc., San Diego, CA, USA).

## Conflict of Interest

Yukimasa Taniguchi declares that the patent holder was one of the inventors of perlecan‐conjugated laminin E8 fragments. Kiyotoshi Sekiguchi declares a leadership position with Matrixome, Inc., one of the inventors of perlecan‐conjugated laminin E8 fragments, and is a co‐founder and shareholder of Matrixome, Inc.

## Author Contributions

The study concept was generated by M.Z., K.S., and H.S., and the experiment was completed by M.Z. with support from T.J., M.N., K.S., and H.S. Microarray analyses were performed by M.Z., T.J., and T.Y. LM‐E8s and NGLFs were prepared by Y.T., C.S., Y.K., and K.S. The mutant bFGFs were supported by Y.C. and M.N. The manuscript and figures were prepared by M.Z. and H.S.

## Supporting information

Supporting Information

## Data Availability

The data that support the findings of this study are openly available in [The DNA Data Bank of Japan] at [https://ddbj.nig.ac.jp/resource/bioproject/PRJDB14707], reference number [14707].
