# Supplementary material for: Heparan Sulfate Chain‐Conjugated Laminin‐E8 Fragments Advance Paraxial Mesodermal Differentiation Followed by High Myogenic Induction from hiPSCs
Source: Adv Sci (Weinh). 2024 Apr 29;11(26):2308306. doi: 10.1002/advs.202308306 (PMC11234437; doi:10.1002/advs.202308306)
Supplement: Supplementary file 1 — Supporting Information [file ADVS-11-2308306-s001.pdf]

ADVANCED SCIENCE
Open Access

Supporting Information

for *Adv. Sci.*, DOI 10.1002/advs.202308306

Heparan Sulfate Chain-Conjugated Laminin-E8 Fragments Advance Paraxial Mesodermal Differentiation Followed by High Myogenic Induction from hiPSCs

Mingming Zhao, Yukimasa Taniguchi, Chisei Shimono, Tatsuya Jonouchi, Yushen Cheng, Yasuhiro Shimizu, Minas Nalbandian, Takuya Yamamoto, Masato Nakagawa, Kiyotoshi Sekiguchi* and Hidetoshi Sakurai**

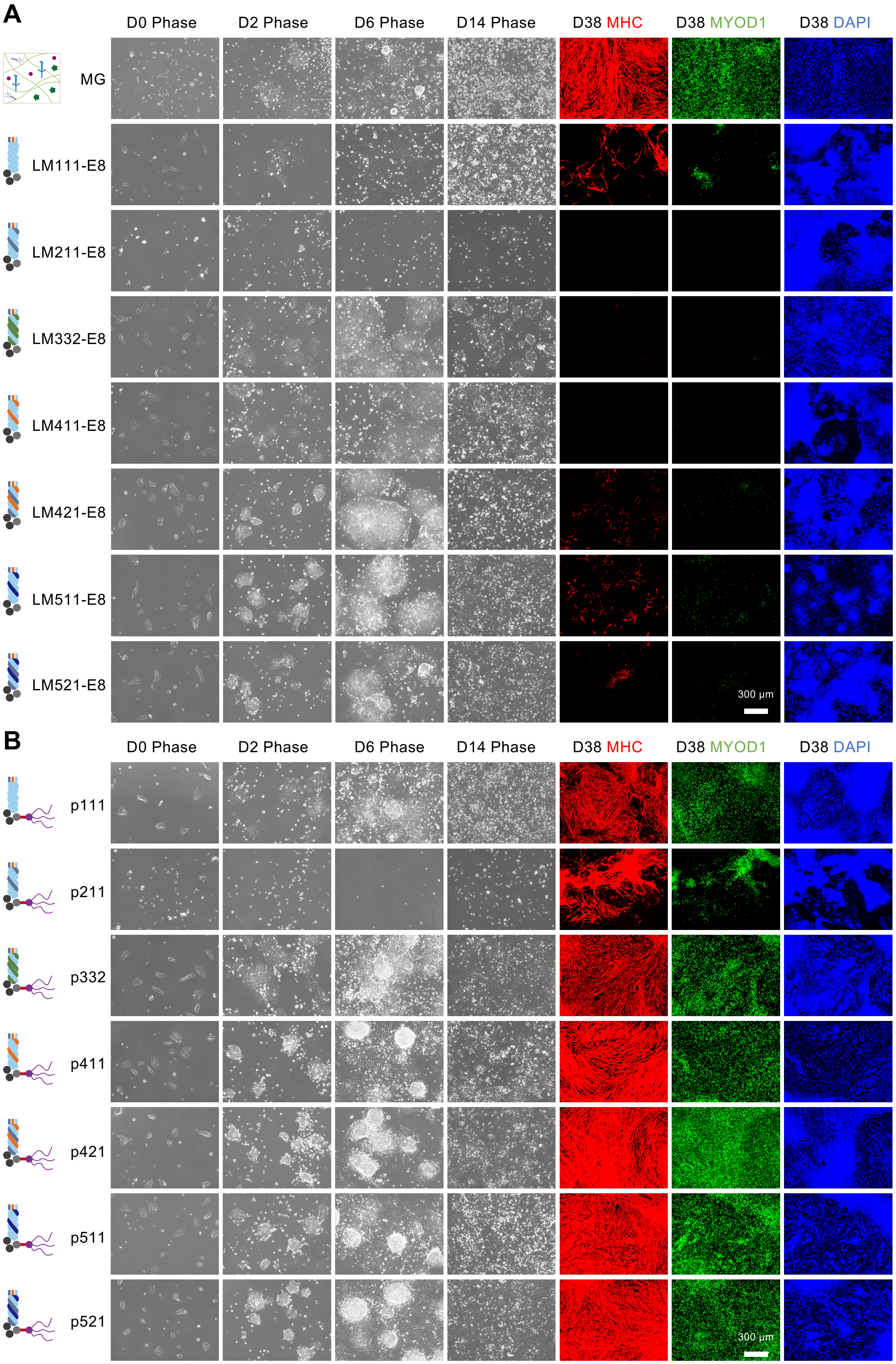

A
D0 Phase
D2 Phase
D6 Phase
D14 Phase
D38 MHC
D38 MYOD1
D38 DAPI
MG
LM111-E8
LM211-E8
LM332-E8
LM411-E8
LM421-E8
LM511-E8
LM521-E8
300 μm
B
D0 Phase
D2 Phase
D6 Phase
D14 Phase
D38 MHC
D38 MYOD1
D38 DAPI
p111
p211
p332
p411
p421
p511
p521
300 μm

Figure S1.

Screening NGLs to optimize myocytes induction from hiPSCs. (A, B) (Left) Schematic representation of the MG, LM-E8s, and NGLFs. **(Middle)** Serial phase-contrast images of hiPSCs (201B7 cell line) on MG, LM-E8s, and NGLFs on days 0 (D0), 2 (D2), 6 (D6), and 14 (D14) of differentiation. LM-E8s support efficient adhesion of hiPSCs, except for LM211-E8 and p211. **(Right)** Immunostaining for myocyte markers in differentiating hiPSCs (201B7 cell line) after 38 days of differentiation on MG, LM-E8s, and NGLs. Myosin heavy chain (MHC, red), MYOD1 (green), and DAPI (blue) staining. Scale bars, 300 μm.

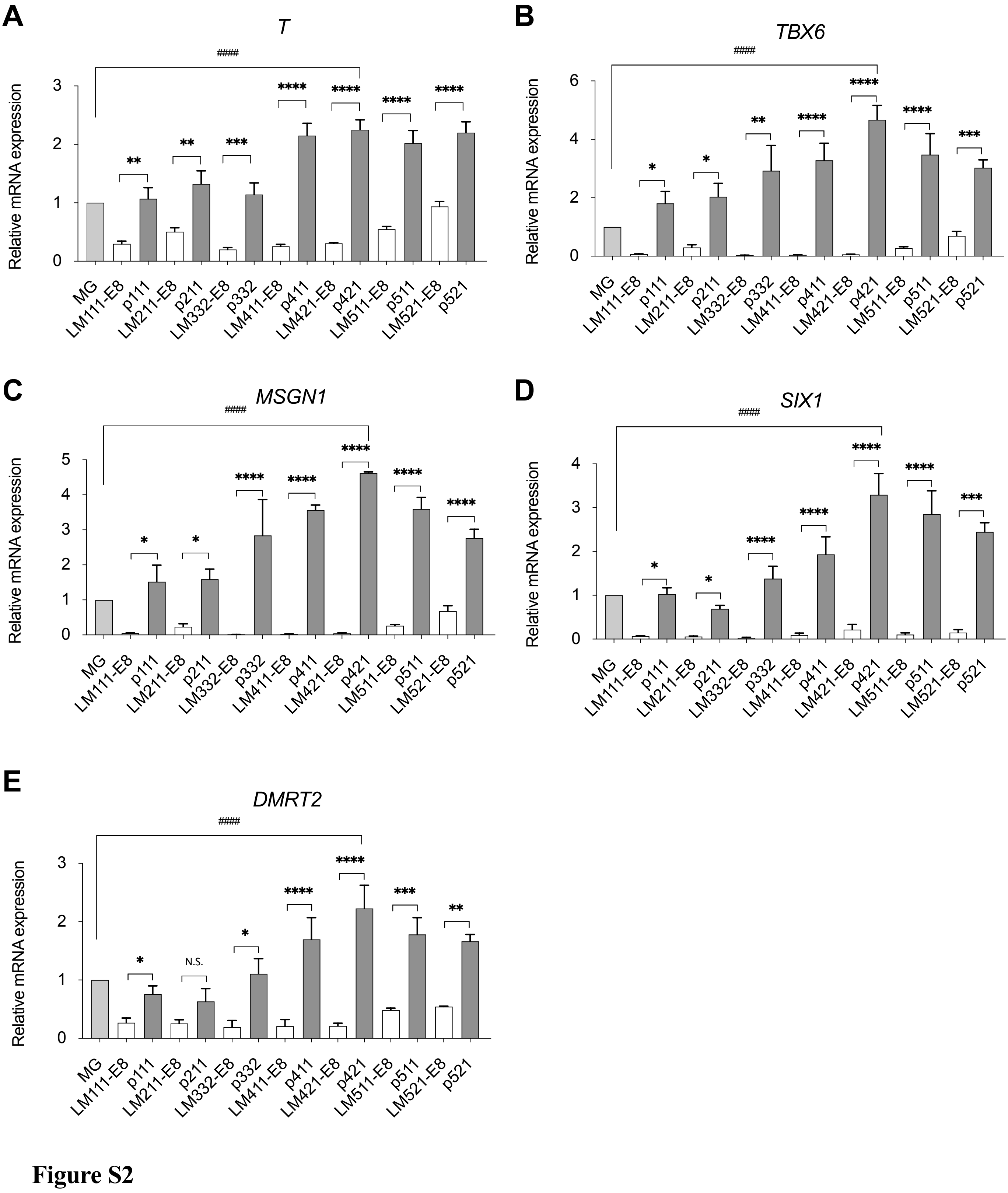

Figure S2

p421 supports efficient myogenic differentiation from hiPSCs. qRT-PCR analysis comparing the expression levels of the primitive streak marker *T* at 2 days of differentiation **(A)**, paraxial mesoderm markers *TBX6* and *MSGN1* after 4 days of differentiation **(B, C)**, and the dermomyotome markers *SIX1* and *DMRT2* at 14 days of differentiation **(D, E)** on MG, LM-E8s, or NGLs. Error bars, mean ± s.d. n = 3. *P*-values were obtained using a one-way ANOVA with Tukey's multiple comparison test. *$P < 0.05$, **$P < 0.01$, ***$P < 0.001$, ****$P < 0.0001$; #$P < 0.05$, ####$P < 0.0001$, N.S.: not significant.

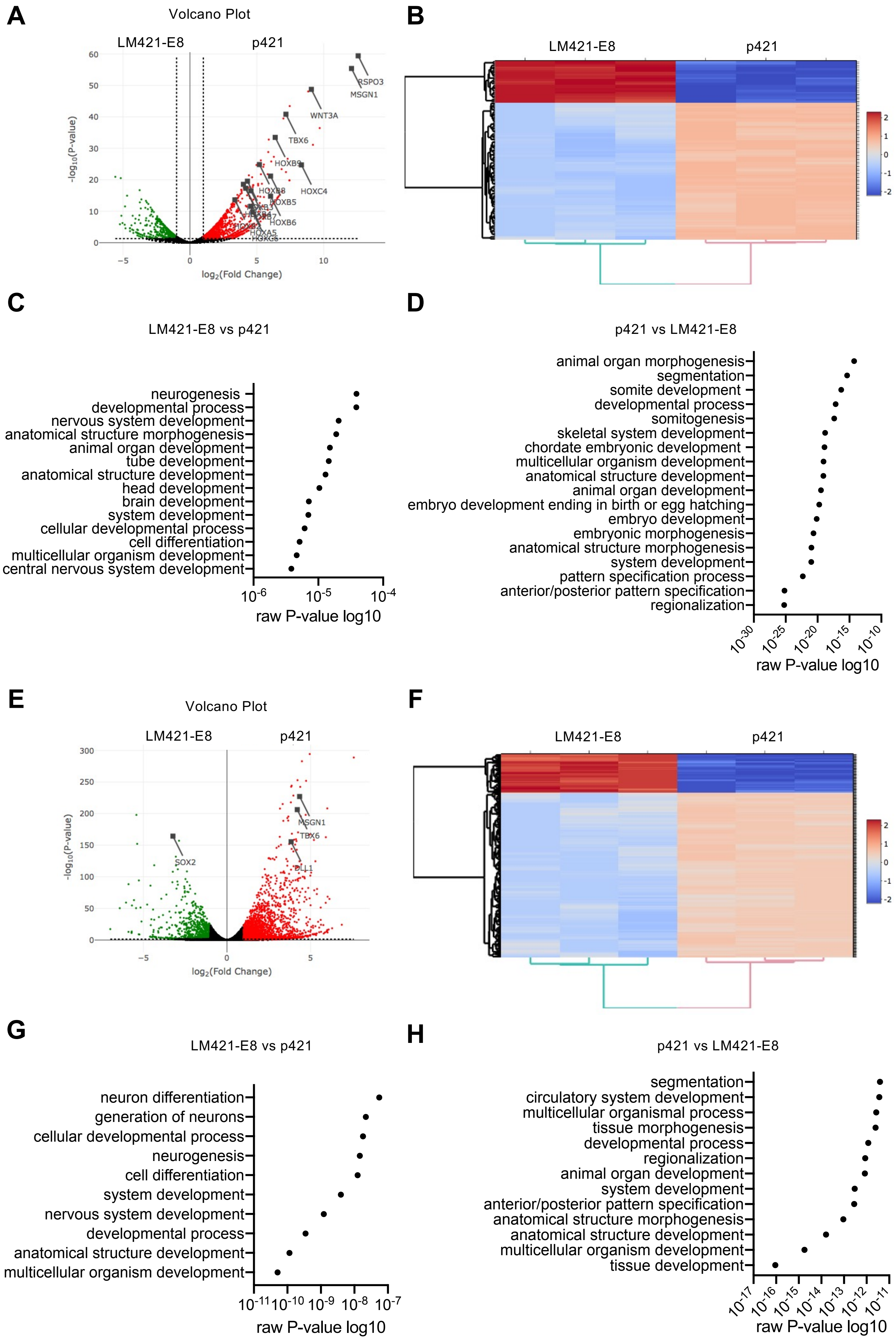
A
Volcano Plot
LM421-E8
p421
-log10(P-value)
log2(Fold Change)
RSPO3
MSGN1
WNT3A
TBX6
HOXB9
HOXC4
HOXB8
HOXB5
HOXB6
HOXA5
B
LM421-E8
p421
C
LM421-E8 vs p421
neurogenesis
developmental process
nervous system development
anatomical structure morphogenesis
animal organ development
tube development
anatomical structure development
head development
brain development
system development
cellular developmental process
cell differentiation
multicellular organism development
central nervous system development
raw P-value log10
D
p421 vs LM421-E8
animal organ morphogenesis
segmentation
somite development
developmental process
somitogenesis
skeletal system development
chordate embryonic development
multicellular organism development
anatomical structure development
animal organ development
embryo development ending in birth or egg hatching
embryo development
embryonic morphogenesis
anatomical structure morphogenesis
system development
pattern specification process
anterior/posterior pattern specification
regionalization
raw P-value log10
E
Volcano Plot
LM421-E8
p421
-log10(P-value)
log2(Fold Change)
MSGN1
TBX6
DLL1
SOX2
F
LM421-E8
p421
G
LM421-E8 vs p421
neuron differentiation
generation of neurons
cellular developmental process
neurogenesis
cell differentiation
system development
nervous system development
developmental process
anatomical structure development
multicellular organism development
raw P-value log10
H
p421 vs LM421-E8
segmentation
circulatory system development
multicellular organismal process
tissue morphogenesis
developmental process
regionalization
animal organ development
system development
anterior/posterior pattern specification
anatomical structure morphogenesis
anatomical structure development
multicellular organism development
tissue development
raw P-value log10

Figure S3

Transcriptomic analysis of differentiated cells cultured in LM421-E8 and p421 on days 2 and 4. (A, B, C, D) Volcano plot **(A)**, heatmap **(B)**, and GO analysis **(C, D)** of differentially expressed genes (DEGs; fold change ≥ 2 and $P < 0.05$) between cells cultured on LM421-E8 and p421 at day 2. **(E, F, G, H)** Volcano plot **(E)**, heatmap **(F)**, and GO analysis **(G, H)** of DEGs (fold change ≥ 2 and $P < 0.05$) between cells cultured on LM421-E8 and p421 at day 4.

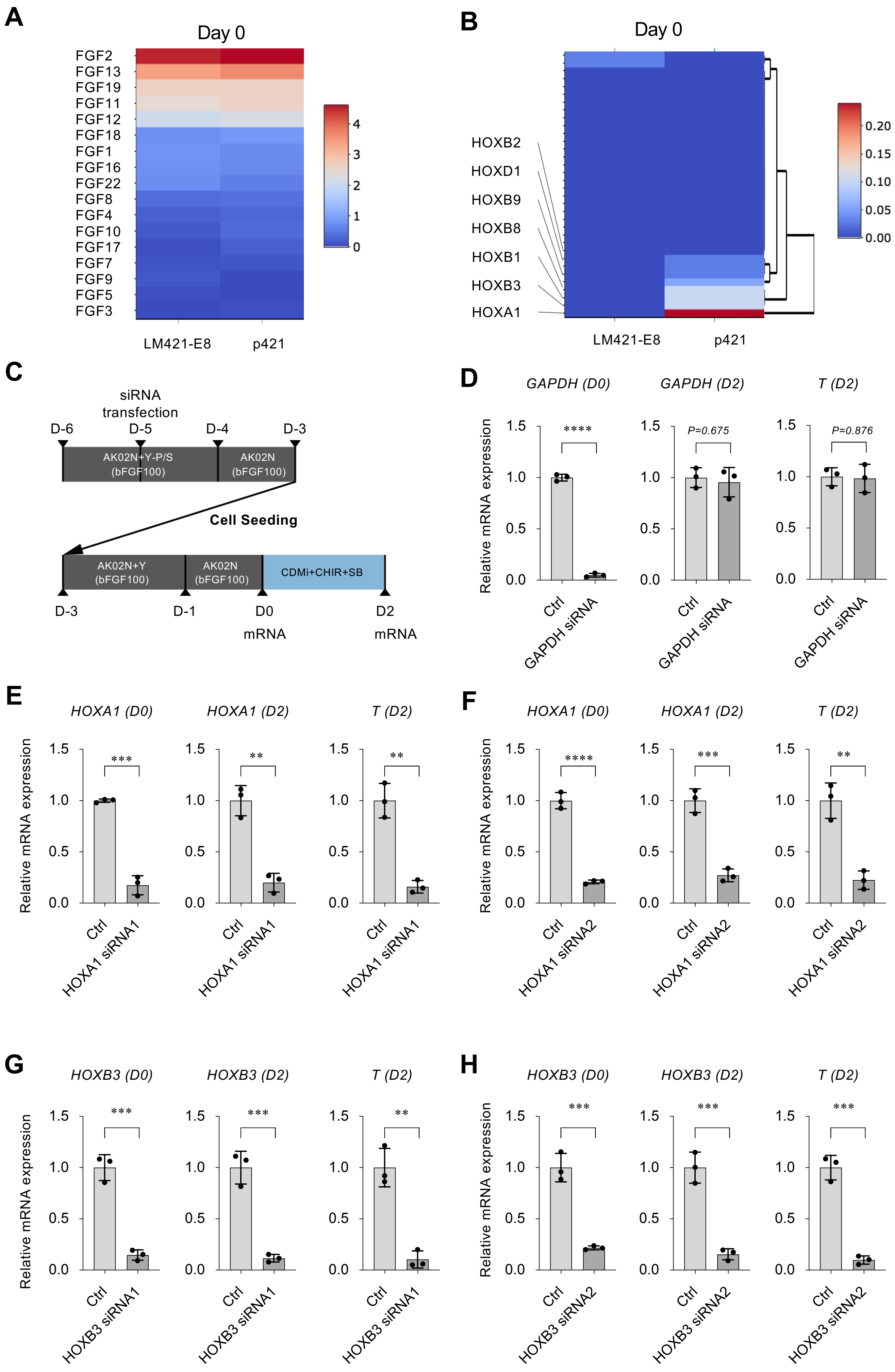

A
Day 0
FGF2
FGF13
FGF19
FGF11
FGF12
FGF18
FGF1
FGF16
FGF22
FGF8
FGF4
FGF10
FGF17
FGF7
FGF9
FGF5
FGF3
LM421-E8
p421
B
Day 0
HOXB2
HOXD1
HOXB9
HOXB8
HOXB1
HOXB3
HOXA1
LM421-E8
p421
C
siRNA transfection
D-6
D-5
D-4
D-3
AK02N+Y-P/S (bFGF100)
AK02N (bFGF100)
Cell Seeding
AK02N+Y (bFGF100)
AK02N (bFGF100)
CDMi+CHIR+SB
D-3
D-1
D0
D2
mRNA
mRNA
D
GAPDH (D0)
GAPDH (D2)
T (D2)
Relative mRNA expression
****
P=0.675
P=0.876
Ctrl
GAPDH siRNA
E
HOXA1 (D0)
HOXA1 (D2)
T (D2)
***
**
**
HOXA1 siRNA1
F
HOXA1 (D0)
HOXA1 (D2)
T (D2)
****
***
**
HOXA1 siRNA2
G
HOXB3 (D0)
HOXB3 (D2)
T (D2)
***
***
**
HOXB3 siRNA1
H
HOXB3 (D0)
HOXB3 (D2)
T (D2)
***
***
***
HOXB3 siRNA2

Figure S4

HOX expression in response to FGF-HS during the pre-culture of hiPSC differentiation. (A) Heat map of normalized gene expression levels for the FGF gene family at day 0. **(B)** Heat map of normalized gene expression levels of the HOX family genes at day 0. **(C)** Schematic illustration of siRNA transfection. siRNAs were transfected 5 days before D0 of differentiation. hiPSCs were transfected with GAPDH, HOXA1, or HOXB3 siRNA**. A**fter two days of transfection (D-3), hiPSCs were seeded on p421-coated dishes for differentiation. mRNA was sampled on D0 and D2 of differentiation. **(D-H)** The mRNA expression of *GAPDH*, *HOXA1*, *HOXB3*, and *T* was analyzed by qRT-PCR on day 0 (D0) or day 2 (D2) of differentiation. Error bars, mean ± s.d. n = 3. *P*-values were obtained using a Student's t-test. $^{**}P < 0.01$, $^{***}P < 0.001$, $^{****}P < 0.0001$.

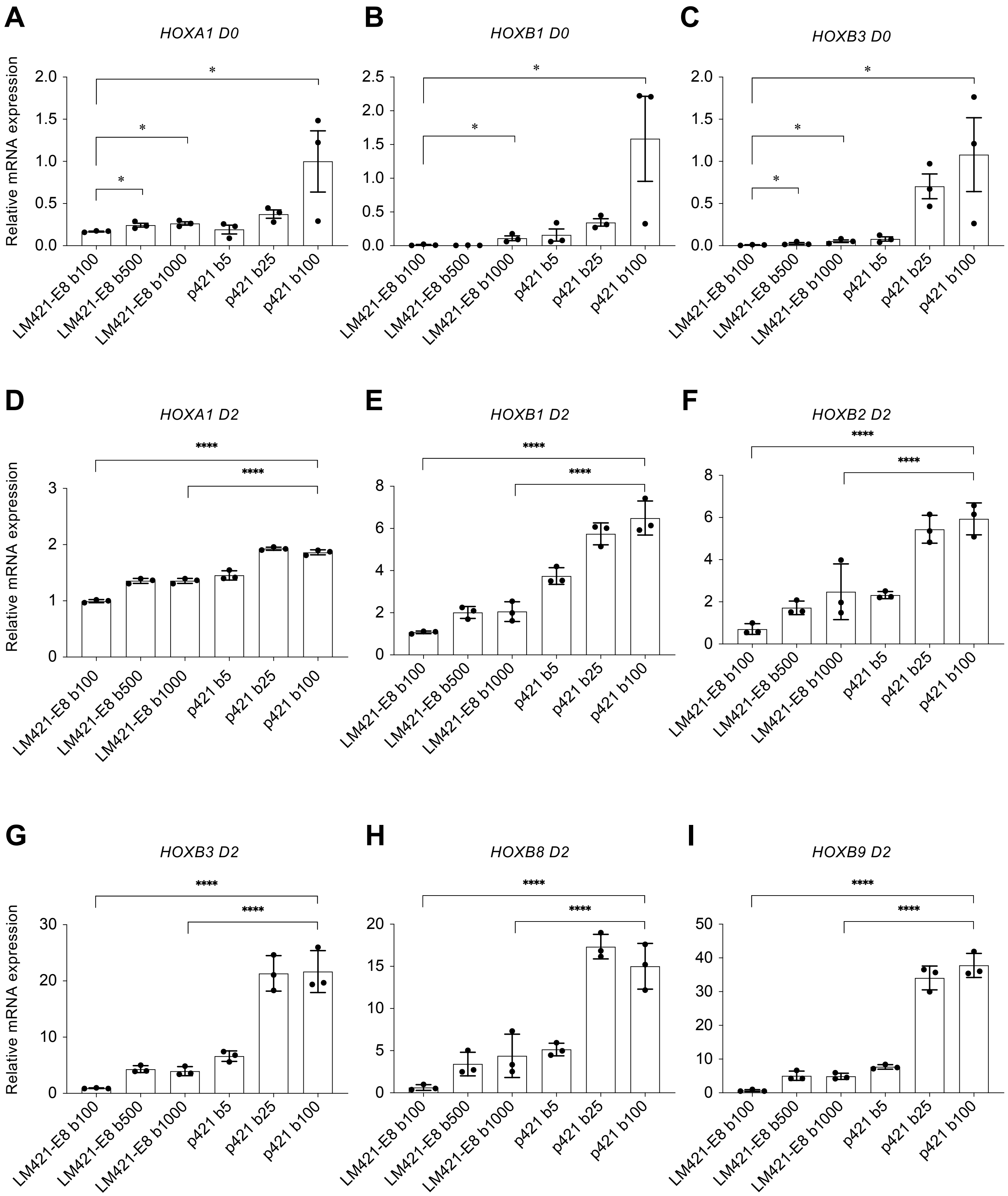

Figure S5

bFGF treatment at the undifferentiated stage increased *HOX* gene expression in a dose-dependent manner. (A-I) hiPSCs were treated with varying levels of bFGF from days -3–0, bFGF dose (b ng mL^{-1}). qRT-PCR analysis indicating the expression level of *HOX* genes on day 0 **(A-C)**, and on day 2 **(D-I)**. Error bars, mean ± s.d. n = 3. *P*-values were obtained using a one-way ANOVA with Tukey's multiple comparison test. *$P < 0.05$, **$P < 0.01$, ***$P < 0.001$, ****$P < 0.0001$.

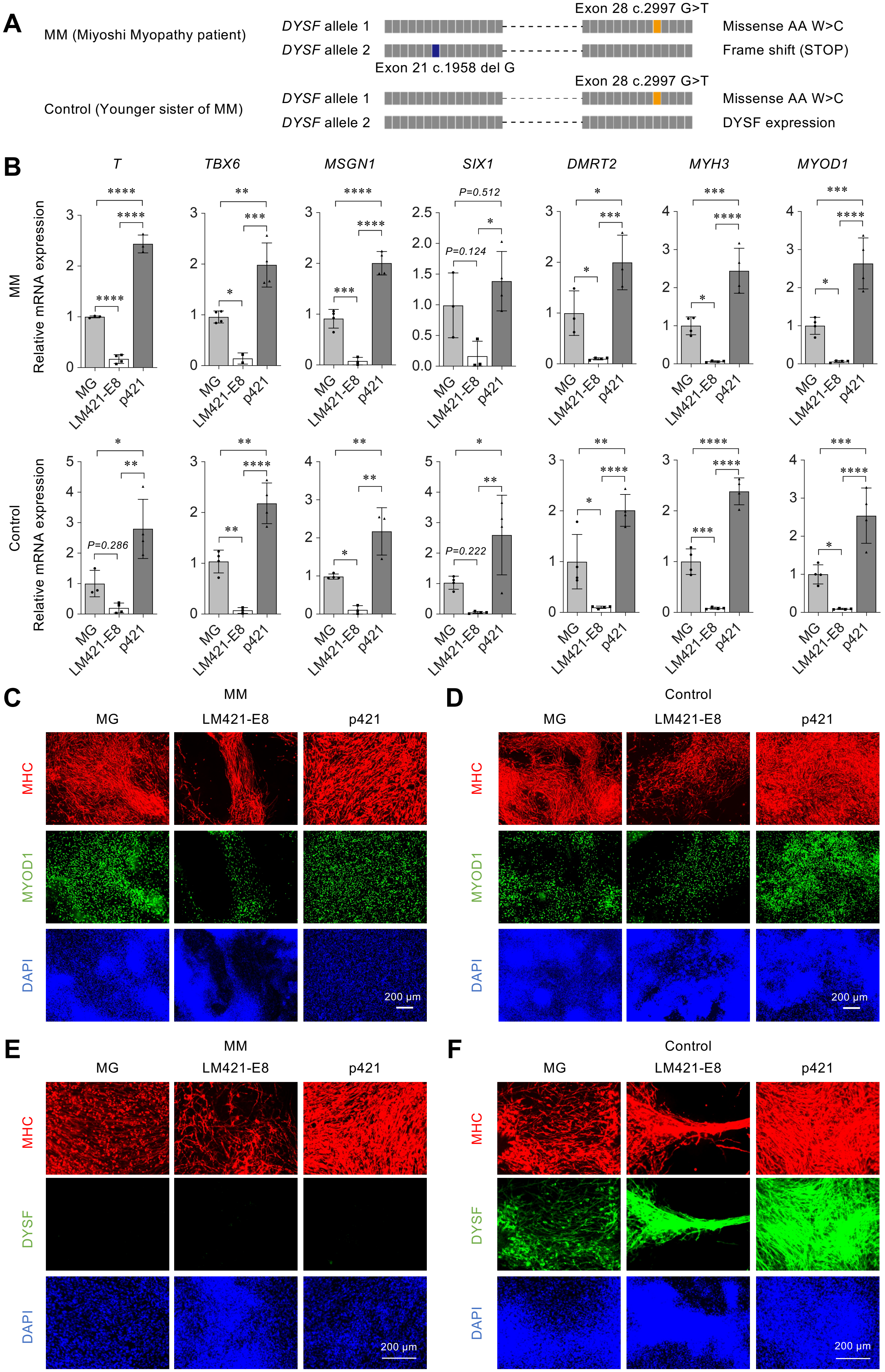
A
MM (Miyoshi Myopathy patient)
DYSF allele 1
DYSF allele 2
Exon 28 c.2997 G>T
Missense AA W>C
Frame shift (STOP)
Exon 21 c.1958 del G
Control (Younger sister of MM)
DYSF allele 1
DYSF allele 2
Exon 28 c.2997 G>T
Missense AA W>C
DYSF expression
B
T
TBX6
MSGN1
SIX1
DMRT2
MYH3
MYOD1
MM
Control
Relative mRNA expression
P=0.512
P=0.124
P=0.286
P=0.222
MG
LM421-E8
p421
C
D
E
F
MM
Control
MHC
MYOD1
DYSF
DAPI
200 μm

Figure S6

Effects of p421 on the myogenic differentiation of Miyoshi myopathy patient-derived iPSCs. **(A)** Schematic illustration of the Miyoshi myopathy patient-derived hiPSC line (MM) and its younger sister-derived control hiPSC line (control). **(B)** qRT-PCR analysis comparing the expression levels of *T* (day 2), *TBX6, MSGN1* (day 4), *SIX1, DMRT2* (day 14), *MYH3,* and *MYOD1* (day 38) on MG, LM-E8s, or p421. Error bars, mean ± s.d. n = 3. *P*-values were obtained using a one-way ANOVA with Tukey's multiple comparison test. $^*P < 0.05$, $^{**}P < 0.01$, $^{***}P < 0.001$, $^{****}P < 0.0001$. **(C, D)** MHC, MYOD1, and DAPI staining of MM- and NOR-derived myocytes (day 38) in MG, LM421-E8, and p421. **(E, F)** MHC, DYSFERLIN (DYSF), and DAPI staining of MM- and control-derived myocytes (day 38) in the MG, LM421-E8, and p421.

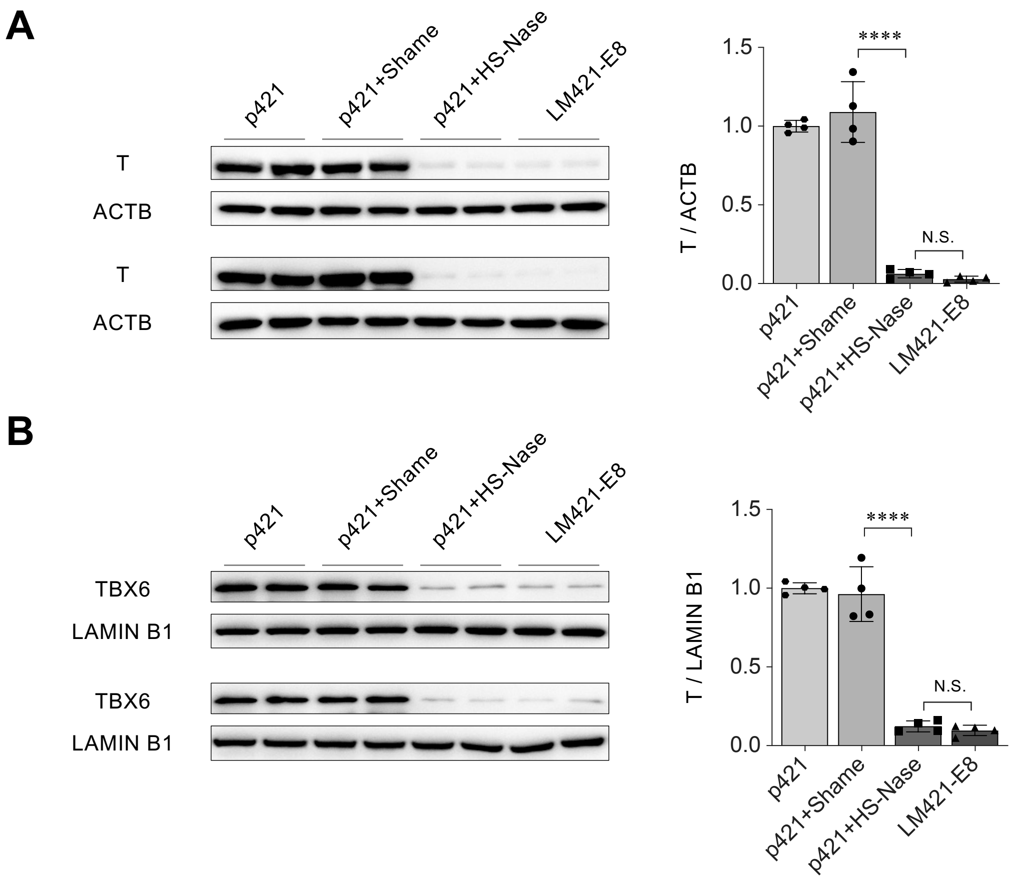

Figure S7

Protein level of T and TBX6 in degradation of HS of p421. (A) Western blot analysis of T (day 2) on p421, p421 + Shame, p421 + HS-Nase, and LM421-E8. ACTB was used as a reference gene. Error bars, mean ± s.d. n = 4. *P*-values were obtained using a one-way ANOVA with Tukey's multiple comparison test. ****$P < 0.0001$, N.S.: not significant. **(B)** Western blot analysis of TBX6 (day 4) on p421, p421 + Shame, p421 + HS-Nase, and LM421-E8. LAMIN B1 was used as reference gene. Error bars, mean ± s.d. n = 4. *P*-values were obtained using a one-way ANOVA with Tukey's multiple comparison test. ****$P < 0.0001$, N.S.: not significant.

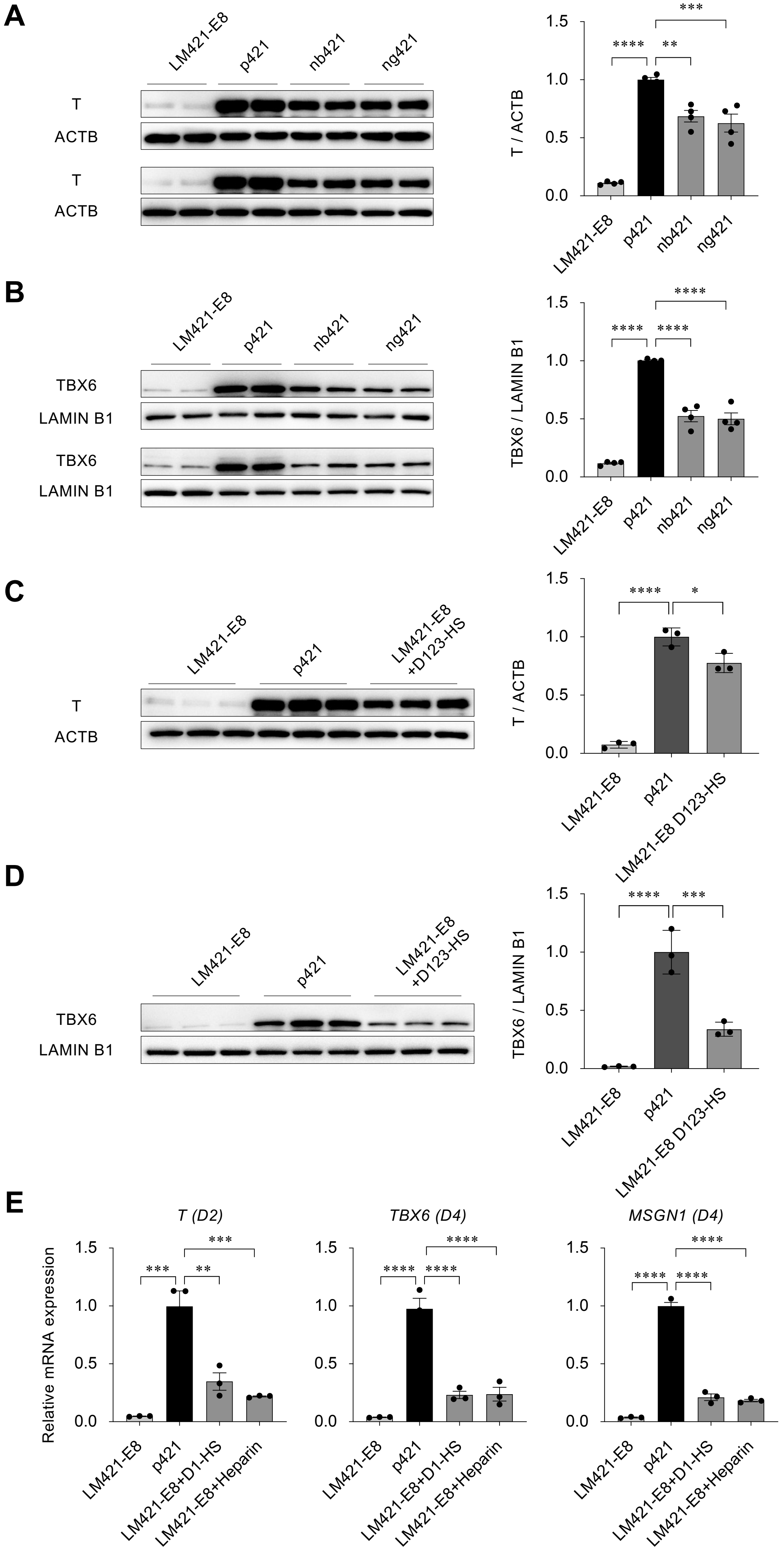
A
LM421-E8
p421
nb421
ng421
T
ACTB
T / ACTB
B
TBX6
LAMIN B1
TBX6 / LAMIN B1
C
LM421-E8 +D123-HS
LM421-E8 D123-HS
D
E
T (D2)
TBX6 (D4)
MSGN1 (D4)
Relative mRNA expression
LM421-E8+D1-HS
LM421-E8+Heparin

Figure S8

Effects of N-terminal HS, D123-HS, D1-HS, and Heparin in hiPSC differentiation. (A) Western blot analysis of T (day 2) on LM421-E8, p421, nb421 (HS conjugated to the N-terminus of the β chain in LM421-E8), and ng421 (HS conjugated to the N-terminus of the γ chain in LM421-E8). ACTB was used as a reference gene. Error bars, mean ± s.d. n = 4. *P*-values were obtained using a one-way ANOVA with Tukey's multiple comparison test. **$P < 0.01$, ***$P < 0.001$, ****$P < 0.0001$. **(B)** Western blot analysis of TBX6 (day 4) on LM421-E8, p421, nb421, and ng421. LAMIN B1 was used as a reference gene. Error bars, mean ± s.d. n = 4. *P*-values were obtained using a one-way ANOVA with Tukey's multiple comparison test. ****$P < 0.0001$. **(C)** Western blot analysis of T (day 2) on LM421-E8, p421, LM421-E8 + D123-HS. ACTB was used as a reference gene. Error bars, mean ± s.d. n = 3. *P*-values were obtained using a one-way ANOVA with Tukey's multiple comparison test. *$P < 0.05$, ****$P < 0.0001$. **(D)** Western blot analysis of TBX6 (day 4) on LM421-E8, p421, LM421-E8 + D123-HS. LAMIN B1 was used as a reference gene. Error bars, mean ± s.d. n = 3. *P*-values were obtained using a one-way ANOVA with Tukey's multiple comparison test. ***$P < 0.001$, ****$P < 0.0001$. **(E)** qRT-PCR analysis comparing the expression levels of *T* (day 2), *TBX6, MSGN1* (day 4) on LM421-E8, p421, LM421-E8 + D1-HS and LM421-E8 + Heparin. Error bars, mean ± s.d. n = 3. *P*-values were obtained using a one-way ANOVA with Tukey's multiple comparison test. **$P < 0.01$, ***$P < 0.001$, ****$P < 0.0001$.

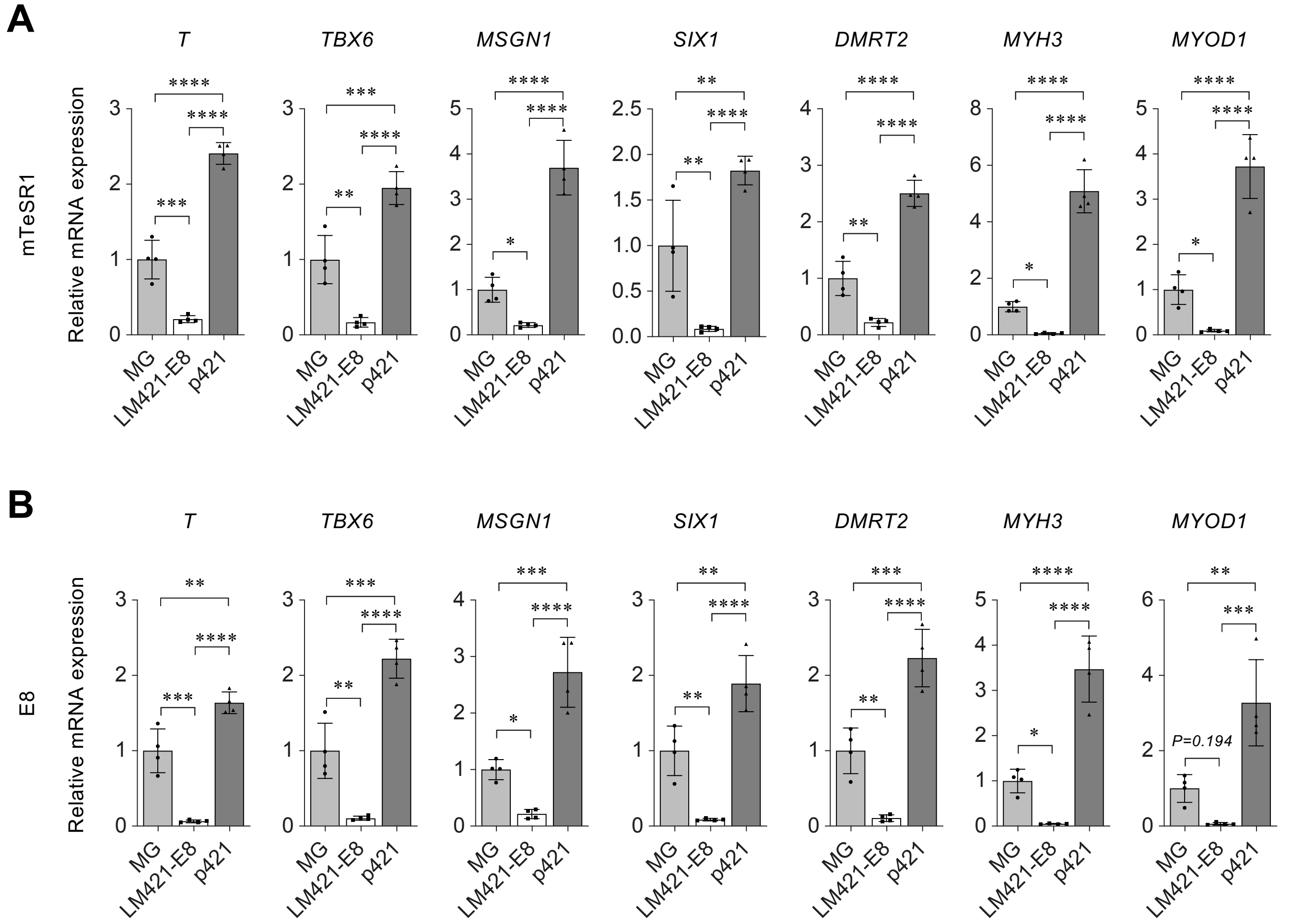

Figure S9

Effects of p421 on the myogenic differentiation in mTeSR1 or E8 cultured hiPSCs. (a) qRT-PCR analysis comparing the expression levels of the primitive streak marker *T* at two days of differentiation, paraxial mesoderm markers *TBX6* and *MSGN1* after four days of differentiation, the dermomyotome markers *SIX1* and *DMRT2* at 14 days of differentiation, and the myocyte markers *MYH3* and *MYOD1* at 38 days of differentiation in mTeSR1 cultured hiPSCs. Error bars, mean ± s.d. n = 4. *P*-values were obtained using a one-way ANOVA with Tukey's multiple comparison test. *$P < 0.05$, **$P < 0.01$, ***$P < 0.001$, ****$P < 0.0001$. (b) **(a)** qRT-PCR analysis comparing the expression levels of the primitive streak marker *T* at two days of differentiation, paraxial mesoderm markers *TBX6* and *MSGN1* after four days of differentiation, the dermomyotome markers *SIX1* and *DMRT2* at 14 days of differentiation, and the myocyte markers *MYH3* and *MYOD1* at 38 days of differentiation in E8 cultured hiPSCs. Error bars, mean ± s.d. n = 4. *P*-values were obtained using a one-way ANOVA with Tukey's multiple comparison test. *$P < 0.05$, **$P < 0.01$, ***$P < 0.001$, ****$P < 0.0001$.

Figure S10
pMAL-c5X-bFGF full vector sequence.

ccgacaccatcgaatggtgcaaaacctttcgcggtatggcatgatagcgcccggaagagagtcaattcagggtggtgaat
gtgaaaccagtaacgttatacgatgtcgcagagtatgccggtgtctcttatcagaccgtttcccgcgtggtgaaccaggcca
gccacgtttctgcgaaaacgcgggaaaaagtggaagcggcgatggcggagctgaattacattcccaaccgcgtggcaca
acaactggcgggcaaacagtcgttgctgattggcgttgccacctccagtctggccctgcacgcgccgtcgcaaattgtcgc
ggcgattaaatctcgcgccgatcaactgggtgccagcgtggtggtgtcgatggtagaacgaagcggcgtcgaagcctgta
aagcggcggtgcacaatcttctcgcgcaacgcgtcagtgggctgatcattaactatccgctggatgaccaggatgccattg
ctgtggaagctgcctgcactaatgttccggcgttatttcttgatgtctctgaccagacacccatcaacagtattattttctcccat
gaagacggtacgcgactgggcgtggagcatctggtcgcattgggtcaccagcaaatcgcgctgttagcgggcccattaag
ttctgtctcggcgcgtctgcgtctggctggctggcataaatatctcactcgcaatcaaattcagccgatagcggaacgggaa
ggcgactggagtgccatgtccggttttcaacaaaccatgcaaatgctgaatgagggcatcgttcccactgcgatgctggttg
ccaacgatcagatggcgctgggcgcaatgcgcgccattaccgagtccgggctgcgcgttggtgcggatatttcggtagtg
ggatacgacgataccgaagacagctcatgttatatcccgccgttaaccaccatcaaacaggattttcgcctgctggggcaaa
ccagcgtggaccgcttgctgcaactctctcagggccaggcggtgaagggcaatcagctgttgcccgtctcactggtgaaa
agaaaaaccaccctggcgcccaatacgcaaaccgcctctccccgcgcgttggccgattcattaatgcagctggcacgaca
ggtttcccgactggaaagcgggcagtgagcgcaacgcaattaatgtaagttagctcactcattaggcacaattctcatgtttg
acagcttatcatcgactgcacggtgcaccaatgcttctggcgtcaggcagccatcggaagctgtggtatggctgtgcaggt
cgtaaatcactgcataattcgtgtcgctcaaggcgcactcccgttctggataatgttttttgcgccgacatcataacggttctgg
caaatattctgaaatgagctgttgacaattaatcatcggctcgtataatgtgtggaattgtgagcggataacaatttcacacag
gaaacagccagtccgtttaggtgttttcacgagcaattgaccaacaaggaccatagattatgaaaatcgaagaaggtaaact
ggtaatctggattaacggcgataaaggctataacggtctcgctgaagtcggtaagaaattcgagaaagataccggaattaa
agtcaccgttgagcatccggataaactggaagagaaattcccacaggttgcggcaactggcgatggccctgacattatcttc
tgggcacacgaccgctttggtggctacgctcaatctggcctgttggctgaaatcaccccggacaaagcgttccaggacaag
ctgtatccgtttacctgggatgccgtacgttacaacggcaagctgattgcttacccgatcgctgttgaagcgttatcgctgattt
ataacaaagatctgctgccgaacccgccaaaaacctgggaagagatcccggcgctggataaagaactgaaagcgaaag
gtaagagcgcgctgatgttcaacctgcaagaaccgtacttcacctggccgctgattgctgctgacgggggttatgcgttcaa
gtatgaaaacggcaagtacgacattaaagacgtgggcgtggataacgctggcgcgaaagcgggtctgaccttcctggttg
acctgattaaaaacaaacacatgaatgcagacaccgattactccatcgcagaagctgcctttaataaaggcgaaacagcga
tgaccatcaacggcccgtgggcatggtccaacatcgacaccagcaaagtgaattatggtgtaacggtactgccgaccttca
agggtcaaccatccaaaccgttcgttggcgtgctgagcgcaggtattaacgccgccagtccgaacaaagagctggcaaaa
gagttcctcgaaaactatctgctgactgatgaaggtctggaagcggttaataaagacaaaccgctgggtgccgtagcgctg
aagtcttacgaggaagagttggtgaaagatccgcgtattgccgccactatggaaaacgcccagaaaggtgaaatcatgcc
gaacatcccgcagatgtccgctttctggtatgccgtgcgtactgcggtgatcaacgccgccagcggtcgtcagactgtcgat
gaagccctgaaagacgcgcagactaattcgagctcgaacaacaacaacaataacaataacaacaacctcgggatcgagg
gaaggatttcacatatgtccatgggcggccgcgatcccgccttgcccgaggatggcggcagcggcgccttcccgcccgg
ccacttcaaggaccccaagcggctgtactgcaaaaacgggggcttcttcctgcgcatccaccccgacggccgagttgacg
gggtccgggagaagagcgaccctcacatcaagctacaacttcaagcagaagagagaggagttgtgtctatcaaaggagt
gtgtgctaaccgttacctggctatgaaggaagatggaagattactggcttctaaatgtgttacggatgagtgtttctttttgaac
gattggaatctaataactacaatacttaccggtcaaggaaatacaccagttggtatgtggcactgaaacgaactgggcagtat
aaacttggatccaaaacaggacctgggcagaaagctatactttttcttccaatgtctgctaagagctgaatcgtcgacggatc
cgaattccctgcaggtaattaaataagcttcaaataaaacgaaaggctcagtcgaaagactgggcctttcgttttatctgttgtt
tgtcggtgaacgctctcctgagtaggacaaatccgccgggagcggatttgaacgttgcgaagcaacggcccggagggtg
gcgggcaggacgcccgccataaactgccaggcatcaaattaagcagaaggccatcctgacggatggcctttttgcgtttct

acaaactctttcggtccgttgtttatttttctaaatacattcaaatatgtatccgctcatgagacaataaccctgataaatgcttcaa
taatattgaaaaaggaagagtatgagtattcaacatttccgtgtcgcccttattcccttttttgcggcattttgccttcctgtttttgc
tcacccagaaacgctggtgaaagtaaaagatgctgaagatcagttgggtgcacgagtgggttacatcgaactggatctcaa
cagcggtaagatccttgagagttttcgccccgaagaacgttttccaatgatgagcacttttaaagttctgctatgtggcgcggt
attatcccgtgttgacgccgggcaagagcaactcggtcgccgcatacactattctcagaatgacttggttgagtactcacca
gtcacagaaaagcatcttacggatggcatgacagtaagagaattatgcagtgctgccataaccatgagtgataacactgcg
gccaacttacttctgacaacgatcggaggaccgaaggagctaaccgcttttttgcacaacatgggggatcatgtaactcgcc
ttgatcgttgggaaccggagctgaatgaagccataccaaacgacgagcgtgacaccacgatgcctgtagcaatggcaaca
acgttgcgcaaactattaactggcgaactacttactctagcttcccggcaacaattaatagactggatggaggcggataaagt
tgcaggaccacttctgcgctcggcccttccggctggctggtttattgctgataaatctggagccggtgagcgtgggtctcgc
ggtatcattgcagcactggggccagatggtaagccctcccgtatcgtagttatctacacgacggggagtcaggcaactatg
gatgaacgaaatagacagatcgctgagataggtgcctcactgattaagcattggtaactgtcagaccaagtttactcatatat
actttagattgatttccttaggactgagcgtcaaccccgtagaaaagatcaaaggatcttcttgagatcctttttttctgcgcgta
atctgctgcttgcaaacaaaaaaaccaccgctaccagcggtggtttgtttgccggatcaagagctaccaactctttttccgaa
ggtaactggcttcagcagagcgcagataccaaatactgtccttctagtgtagccgtagttaggccaccacttcaagaactctg
tagcaccgcctacatacctcgctctgctaatcctgttaccagtggctgctgccagtggcgataagtcgtgtcttaccgggttg
gactcaagacgatagttaccggataaggcgcagcggtcgggctgaacggggggttcgtgcacacagcccagcttggag
cgaacgacctacaccgaactgagatacctacagcgtgagctatgagaaagcgccacgcttcccgaagggagaaaggcg
gacaggtatccggtaagcggcagggtcggaacaggagagcgcacgagggagcttccagggggaaacgcctggtatctt
tatagtcctgtcgggtttcgccacctctgacttgagcgtcgatttttgtgatgctcgtcaggggggcggagcctatggaaaaa
cgccagcaacgcggcctttttacggttcctggccttttgctggccttttgctcacatgttctttcctgcgttatcccctgattctgt
ggataaccgtattaccgcctttgagtgagctgataccgctcgccgcagccgaacgaccgagcgcagcgagtcagtgagc
gaggaagcggaagagcgcctgatgcggtattttctccttacgcatctgtgcggtatttcacaccgcatataaggtgcactgtg
actgggtcatggctgcgccccgacacccgccaacacccgctgacgcgccctgacgggcttgtctgctcccggcatccgct
tacagacaagctgtgaccgtctccgggagctgcatgtgtcagaggttttcaccgtcatcaccgaaacgcgcgaggcagct
gcggtaaagctcatcagcgtggtcgtgcagcgattcacagatgtctgcctgttcatccgcgtccagctcgttgagtttctcca
gaagcgttaatgtctggcttctgataaagcgggccatgttaagggcggttttttcctgtttggtcactgatgcctccgtgtaagg
gggatttctgttcatgggggtaatgataccgatgaaacgagagaggatgctcacgatacgggttactgatgatgaacatgcc
cggttactggaacgttgtgagggtaaacaactggcggtatggatgcggcgggaccagagaaaaatcactcagggtcaatg
ccagcgcttcgttaatacagatgtaggtgttccacagggtagccagcagcatcctgcgatgcagatccggaacataatggt
gcagggcgctgacttccgcgtttccagactttacgaaacacggaaaccgaagaccattcatgttgttgctcaggtcgcagac
gttttgcagcagcagtcgcttcacgttcgctcgcgtatcggtgattcattctgctaaccagtaaggcaaccccgccagcctag
ccgggtcctcaacgacaggagcacgatcatgcgcacccgtggccaggacccaacgctgcccgaaatt

Figure S11

cDNA sequences of LMα4-E8 conjugated with the D1 domain of perlecan, LMβ2-E8, and LMγ1-E8

> P-α4E8 (3,045 bp)

```
  1   ATGGAGACAGACACACTCCTGCTATGGGTACTGCTGCTCTGGGTTCCAGGTTCCACTGGT
  1   -M--E--T--D--T--L--L--L--W--V--L--L--L--W--V--P--G--S--T--G-

 61   GACGCGGCCCAGCCGGCCAGGCGCGCGCCGTACGAAGCTTCATCATCATCATCATCAT
 21   -D--A--A--Q--P--A--R--R--A--R--R--T--K--L--H--H--H--H--H--H-

121   GATGAAGCCAATGAAACAGCAGAATTTGCTTTGAACACCACTGACCGAATTTATGATGCG
 41   -D--E--A--N--E--T--A--E--F--A--L--N--T--T--D--R--I--Y--D--A-

181   GTGAGTGGGATTGATACTCAAATCATTTACCATAAAGATGAAAGTGAGAACCTCCTCAAT
 61   -V--S--G--I--D--T--Q--I--I--Y--H--K--D--E--S--E--N--L--L--N-

241   CAAGCCAGAGAACTGCAAGCAAAGGCAGAGTCTAGCAGTGATGAAGCAGTGGCTGACACT
 81   -Q--A--R--E--L--Q--A--K--A--E--S--S--S--D--E--A--V--A--D--T-

301   AGCAGGCGTGTGGGTGGAGCCCTAGCAAGGAAAAGTGCCCTTAAAACCAGACTCAGTGAT
101   -S--R--R--V--G--G--A--L--A--R--K--S--A--L--K--T--R--L--S--D-

361   GCCGTTAAGCAACTACAAGCAGCAGAGAGAGGGGATGCCCAGCAGCGCCTGGGGCAGTCT
121   -A--V--K--Q--L--Q--A--A--E--R--G--D--A--Q--Q--R--L--G--Q--S-

421   AGACTGATCACCGAGGAAGCCAACAGGACGACGATGGAGGTGCAGCAGGCCACTGCCCCC
141   -R--L--I--T--E--E--A--N--R--T--T--M--E--V--Q--Q--A--T--A--P-

481   ATGGCCAACAATCTAACCAACTGGTCACAGAATCTTCAACATTTTGACTCTTCTGCTTAC
161   -M--A--N--N--L--T--N--W--S--Q--N--L--Q--H--F--D--S--S--A--Y-

541   AACACTGCAGTGAACTCTGCTAGGGATGCAGTAAGAAATCTGACCGAGGTTGTCCCTCAG
181   -N--T--A--V--N--S--A--R--D--A--V--R--N--L--T--E--V--V--P--Q-

601   CTCCTGGATCAGCTTCGTACGGTTGAGCAGAAGCGACCTGCAAGCAACGTTTCTGCCAGC
201   -L--L--D--Q--L--R--T--V--E--Q--K--R--P--A--S--N--V--S--A--S-

661   ATCCAGAGGATCCGAGAGCTCATTGCTCAGACCAGAAGTGTTGCCAGCAAGATCCAAGTC
221   -I--Q--R--I--R--E--L--I--A--Q--T--R--S--V--A--S--K--I--Q--V-
```

```
 721  TCCATGATGTTTGATGGCCAGTCAGCTGTGGAAGTGCACTCGAGAACCAGTATGGATGAC
 241  -S--M--M--F--D--G--Q--S--A--V--E--V--H--S--R--T--S--M--D--D-
 781  TTAAAGGCCTTCACGTCTCTGAGCCTGTACATGAAACCCCCTGTGAAGCGGCCGGAACTG
 261  -L--K--A--F--T--S--L--S--L--Y--M--K--P--P--V--K--R--P--E--L-

 841  ACCGAGACTGCAGATCAGTTTATCCTGTACCTCGGAAGCAAAAACGCCAAAAAAGAGTAT
 281  -T--E--T--A--D--Q--F--I--L--Y--L--G--S--K--N--A--K--K--E--Y-

 901  ATGGGTCTTGCAATCAAAAATGATAATCTGGTATACGTCTATAATTTGGGAACTAAAGAT
 301  -M--G--L--A--I--K--N--D--N--L--V--Y--V--Y--N--L--G--T--K--D-

 961  GTGGAGATTCCCCTGGACTCCAAGCCCGTCAGTTCCTGGCCTGCTTACTTCAGCATTGTC
 321  -V--E--I--P--L--D--S--K--P--V--S--S--W--P--A--Y--F--S--I--V-

1021  AAGATTGAAAGGGTGGGAAAACATGGAAAGGTGTTTTTAACAGTCCCGAGTCTAAGTAGC
 341  -K--I--E--R--V--G--K--H--G--K--V--F--L--T--V--P--S--L--S--S-

1081  ACAGCAGAGGAAAAGTTCATTAAAAAGGGGGAATTTTCGGGAGATGACTCTCTGCTGGAC
 361  -T--A--E--E--K--F--I--K--K--G--E--F--S--G--D--D--S--L--L--D-

1141  CTGGACCCTGAGGACACAGTGTTTTATGTTGGTGGAGTGCCTTCCAACTTCAAGCTCCCT
 381  -L--D--P--E--D--T--V--F--Y--V--G--G--V--P--S--N--F--K--L--P-

1201  ACCAGCTTAAACCTGCCTGGCTTTGTTGGCTGCCTGGAACTGGCCACTTTGAATAATGAT
 401  -T--S--L--N--L--P--G--F--V--G--C--L--E--L--A--T--L--N--N--D-

1261  GTGATCAGCTTGTACAACTTTAAGCACATCTATAATATGGACCCCTCCACATCAGTGCCA
 421  -V--I--S--L--Y--N--F--K--H--I--Y--N--M--D--P--S--T--S--V--P-

1321  TGTGCCCGAGATAAGCTGGCCTTCACTCAGAGTCGGGCTGCCAGTTACTTCTTCGATGGC
 441  -C--A--R--D--K--L--A--F--T--Q--S--R--A--A--S--Y--F--F--D--G-

1381  TCCGGTTATGCCGTGGTGAGAGACATCACAAGGAGAGGGAAATTTGGTCAGGTGACTCGC
 461  -S--G--Y--A--V--V--R--D--I--T--R--R--G--K--F--G--Q--V--T--R-

1441  TTTGACATAGAAGTTCGAACACCAGCTGACAACGGCCTTATTCTCCTGATGGTCAATGGA
 481  -F--D--I--E--V--R--T--P--A--D--N--G--L--I--L--L--M--V--N--G-

1501  AGTATGTTTTTCAGACTGGAAATGCGCAATGGTTACCTACATGTGTTCTATGATTTTGGA
 501  -S--M--F--F--R--L--E--M--R--N--G--Y--L--H--V--F--Y--D--F--G-
```

1561 TTCAGCGGTGGCCCTGTGCATCTTGAAGATACGTTAAAGAAAGCTCAAATTAATGATGCA
521 -F--S--G--G--P--V--H--L--E--D--T--L--K--K--A--Q--I--N--D--A-

1621 AAATACCATGAGATCTCAATCATTTACCACAATGATAAGAAAATGATCTTGGTAGTTGAC
541 -K--Y--H--E--I--S--I--I--Y--H--N--D--K--K--M--I--L--V--V--D-

1681 AGAAGGCATGTCAAGAGCATGGATAATGAAAAGATGAAAATACCTTTTACAGATATATAC
561 -R--R--H--V--K--S--M--D--N--E--K--M--K--I--P--F--T--D--I--Y-

1741 ATTGGAGGAGCTCCTCCAGAAATCTTACAATCCAGGGCCCTCAGAGCACACCTTCCCCTA
581 -I--G--G--A--P--P--E--I--L--Q--S--R--A--L--R--A--H--L--P--L-

1801 GATATCAACTTCAGAGGATGCATGAAGGGCTTCCAGTTCCAAAAGAAGGACTTCAATTTA
601 -D--I--N--F--R--G--C--M--K--G--F--Q--F--Q--K--K--D--F--N--L-

1861 CTGGAGCAGACAGAAACCCTGGGAGTTGGTTATGGATGCCCAGAAGACTCACTTATATCT
621 -L--E--Q--T--E--T--L--G--V--G--Y--G--C--P--E--D--S--L--I--S-

1921 CGCAGAGCATATTTCAATGGACAGAGCTTCATTGCTTCAATTCAGAAAATATCTTTCTTT
641 -R--R--A--Y--F--N--G--Q--S--F--I--A--S--I--Q--K--I--S--F--F-

1981 GATGGCTTTGAAGGAGGTTTTAATTTCCGAACATTACAACCAAATGGGTTACTATTCTAT
661 -D--G--F--E--G--G--F--N--F--R--T--L--Q--P--N--G--L--L--F--Y-

2041 TATGCTTCAGGGTCAGACGTGTTCTCCATCTCACTGGATAATGGTACTGTCATCATGGAT
681 -Y--A--S--G--S--D--V--F--S--I--S--L--D--N--G--T--V--I--M--D-

2101 GTAAAGGGAATCAAAGTTCAGTCAGTAGATAAGCAGTACAATGATGGGCTGTCCCACTTC
701 -V--K--G--I--K--V--Q--S--V--D--K--Q--Y--N--D--G--L--S--H--F-

2161 GTCATTAGCTCTGTCTCACCCACAAGATATGAACTGATAGTAGATAAAAGCAGAGTTGGG
721 -V--I--S--S--V--S--P--T--R--Y--E--L--I--V--D--K--S--R--V--G-

2221 AGTAAGAATCCTACCAAAGGGAAAATAGAACAGACACAAGCAAGTGAAAAGAAGTTTTAC
741 -S--K--N--P--T--K--G--K--I--E--Q--T--Q--A--S--E--K--K--F--Y-

2281 TTCGGTGGCTCACCAATCAGTGCTCAGTATGCTAATTTCACTGGCTGCATAAGTAATGCC
761 -F--G--G--S--P--I--S--A--Q--Y--A--N--F--T--G--C--I--S--N--A-

```
2341   TACTTTACCAGGGTGGATAGAGATGTGGAGGTTGAAGATTTCCAACGGTATACTGAAAAG
 781   -Y--F--T--R--V--D--R--D--V--E--V--E--D--F--Q--R--Y--T--E--K-

2401   GTCCACACTTCTCTTTATGAGTGTCCCATTGAGTCTTCACCATTGTTTCTCCTCCATGAT
 801   -V--H--T--S--L--Y--E--C--P--I--E--S--S--P--L--F--L--L--H--D-

2461   GCAGAGGACAGCAAGCTCTTGCCAGAGCCCCGGGCTTTTCCAGGATCCGGGCTGAGGGCA
 821   -A--E--D--S--K--L--L--P--E--P--R--A--F--P--G--S--G--L--R--A-

2521   TACGATGGCTTGTCTCTGCCTGAGGACATAGAGACCGTCACAGCAAGCCAAATGCGCTGG
 841   -Y--D--G--L--S--L--P--E--D--I--E--T--V--T--A--S--Q--M--R--W-

2581   ACACATTCGTACCTTTCTGATGATGAGGACATGCTGGCTGACAGCATCTCAGGAGACGAC
 861   -T--H--S--Y--L--S--D--D--E--D--M--L--A--D--S--I--S--G--D--D-

2641   CTGGGCAGTGGGGACCTGGGCAGCGGGGACTTCCAGATGGTTTATTTCCGAGCCCTGGTG
 881   -L--G--S--G--D--L--G--S--G--D--F--Q--M--V--Y--F--R--A--L--V-

2701   AATTTCACTCGCTCCATCGAGTACAGCCCTCAGCTGGAGGATGCAGGCTCCAGAGAGTTC
 901   -N--F--T--R--S--I--E--Y--S--P--Q--L--E--D--A--G--S--R--E--F-

2761   CGAGAGGTGTCCGAGGCTGTGGTAGACACGCTGGAGTCGGAGTACTTGAAAATTCCCGGA
 921   -R--E--V--S--E--A--V--V--D--T--L--E--S--E--Y--L--K--I--P--G-

2821   GACCAGGTTGTCAGTGTGGTGTTCATCAAGGAGCTGGATGGCTGGGTTTTTGTGGAGCTG
 941   -D--Q--V--V--S--V--V--F--I--K--E--L--D--G--W--V--F--V--E--L-

2881   GATGTGGGCTCGGAAGGGAATGCGGATGGGGCTCAGATTCAGGAGATGCTGCTCAGGGTC
 961   -D--V--G--S--E--G--N--A--D--G--A--Q--I--Q--E--M--L--L--R--V-

2941   ATCTCCAGCGGCTCTGTGGCCTCCTACGTCACCTCTCCCCAGGGATTCCAGTTCCGACGC
 981   -I--S--S--G--S--V--A--S--Y--V--T--S--P--Q--G--F--Q--F--R--R-

3001   CTGGGCACAGTGCCCCAGTTCCCACATCATCATCATCATCATTAG
1001   -L--G--T--V--P--Q--F--P--H--H--H--H--H--H--*-
```

- Red letters, A linker sequence of human laminin α1
- Blue letters, human perlecan D1 domain

> LMβ2E8 (819 bp)

```
  1  ATGGAGACAGACACACTCCTGCTATGGGTACTGCTGCTCTGGGTTCCAGGTTCCACTGGT
  1  -M--E--T--D--T--L--L--L--W--V--L--L--L--W--V--P--G--S--T--G-

 61  GACGCGGCCCAGCCGGCCAGGCGCGCGCCGTACGAAGCTTGGTACCTATCCATATGAT
 21  -D--A--A--Q--P--A--R--R--A--R--R--T--K--L--G--T--Y--P--Y--D-

121  GTGCCAGATTATGCAGATCTGGCACGTACTGTAGGAGATGTGCGTCGTGCCGAGCAGCTA
 41  -V--P--D--Y--A--D--L--A--R--T--V--G--D--V--R--R--A--E--Q--L-

181  CTGCAGGATGCACGGCGGGCAAGGAGCTGGGCTGAGGATGAGAAACAGAAGGCAGAGACA
 61  -L--Q--D--A--R--R--A--R--S--W--A--E--D--E--K--Q--K--A--E--T-

241  GTACAGGCAGCACTGGAGGAGGCCCAGCGGGCACAGGGTATTGCCCAGGGTGCCATCCGG
 81  -V--Q--A--A--L--E--E--A--Q--R--A--Q--G--I--A--Q--G--A--I--R-

301  GGGGCAGTGGCTGACACACGGGACACAGAGCAGACCCTGTACCAGGTACAGGAGAGGATG
101  -G--A--V--A--D--T--R--D--T--E--Q--T--L--Y--Q--V--Q--E--R--M-

361  GCAGGTGCAGAGCGGGCACTGAGCTCTGCAGGTGAAAGGGCTCGGCAGTTGGATGCTCTC
121  -A--G--A--E--R--A--L--S--S--A--G--E--R--A--R--Q--L--D--A--L-

421  CTGGAGGCTCTGAAATTGAAACGGGCAGGAAATAGTCTGGCAGCCTCTACAGCAGAAGAA
141  -L--E--A--L--K--L--K--R--A--G--N--S--L--A--A--S--T--A--E--E-

481  ACGGCAGGCAGTGCCCAGGGTCGTGCCCAGGAGGCTGAGCAGCTGCTACGCGGTCCTCTG
161  -T--A--G--S--A--Q--G--R--A--Q--E--A--E--Q--L--L--R--G--P--L-

541  GGTGATCAGTACCAGACGGTGAAGGCCCTAGCTGAGCGCAAGGCCCAAGGTGTGCTGGCT
181  -G--D--Q--Y--Q--T--V--K--A--L--A--E--R--K--A--Q--G--V--L--A-

601  GCACAGGCAAGGGCAGAACAACTGCGGGATGAGGCTCGGGACCTGTTGCAAGCCGCTCAG
201  -A--Q--A--R--A--E--Q--L--R--D--E--A--R--D--L--L--Q--A--A--Q-

661  GACAAGCTGCAGCGGCTACAGGAATTGGAAGGCACCTATGAGGAAAATGAGCGGGCACTG
221  -D--K--L--Q--R--L--Q--E--L--E--G--T--Y--E--E--N--E--R--A--L-

721  GAGAGTAAGGCAGCCCAGTTGGACGGGTTGGAGGCCAGGATGCGCAGCGTGCTTCAAGCC
241  -E--S--K--A--A--Q--L--D--G--L--E--A--R--M--R--S--V--L--Q--A-
```

781   ATCAACTTGCAGGTGCAGATCTACAACACCTGCCAGTGA
261   -I--N--L--Q--V--Q--I--Y--N--T--C--Q--*-

```
> LMγ1E8 (870 bp)

  1  ATGGAGACAGACACACTCCTGCTATGGGTACTGCTGCTCTGGGTTCCAGGTTCCACTGGT
  1  -M--E--T--D--T--L--L--L--W--V--L--L--L--W--V--P--G--S--T--G-

 61  GACGCGGCCCAGCCGGCCAGGCGCGCGCCGTACGAAGCTTGATTACAAGGATGATGAT
 21  -D--A--A--Q--P--A--R--R--A--R--R--T--K--L--D--Y--K--D--D--D-

121  GATAAGGATAATGACATTCTCAACAACCTGAAAGATTTTGATAGGCGCGTGAACGATAAC
 41  -D--K--D--N--D--I--L--N--N--L--K--D--F--D--R--R--V--N--D--N-

181  AAGACGGCCGCAGAGGAGGCACTAAGGAAGATTCCTGCCATCAACCAGACCATCACTGAA
 61  -K--T--A--A--E--E--A--L--R--K--I--P--A--I--N--Q--T--I--T--E-

241  GCCAATGAAAAGACCAGAGAAGCCCAGCAGGCCCTGGGCAGTGCTGCGGCGGATGCCACA
 81  -A--N--E--K--T--R--E--A--Q--Q--A--L--G--S--A--A--A--D--A--T-

301  GAGGCCAAGAACAAGGCCCATGAGGCGGAGAGGATCGCAAGCGCTGTCCAAAAGAATGCC
101  -E--A--K--N--K--A--H--E--A--E--R--I--A--S--A--V--Q--K--N--A-

361  ACCAGCACCAAGGCAGAAGCTGAAAGAACTTTTGCAGAAGTTACAGATCTGGATAATGAG
121  -T--S--T--K--A--E--A--E--R--T--F--A--E--V--T--D--L--D--N--E-

421  GTGAACAATATGTTGAAGCAACTGCAGGAAGCAGAAAAAGAGCTAAAGAGAAAACAAGAT
141  -V--N--N--M--L--K--Q--L--Q--E--A--E--K--E--L--K--R--K--Q--D-

481  GACGCTGACCAGGACATGATGATGGCAGGGATGGCTTCACAGGCTGCTCAAGAAGCCGAG
161  -D--A--D--Q--D--M--M--M--A--G--M--A--S--Q--A--A--Q--E--A--E-

541  ATCAATGCCAGAAAAGCCAAAAACTCTGTTACTAGCCTCCTCAGCATTATTAATGACCTC
181  -I--N--A--R--K--A--K--N--S--V--T--S--L--L--S--I--I--N--D--L-

601  TTGGAGCAGCTGGGGCAGCTGGATACAGTGGACCTGAATAAGCTAAACGAGATTGAAGGC
201  -L--E--Q--L--G--Q--L--D--T--V--D--L--N--K--L--N--E--I--E--G-

661  ACCCTAAACAAAGCCAAAGATGAAATGAAGGTCAGCGATCTTGATAGGAAAGTGTCTGAC
221  -T--L--N--K--A--K--D--E--M--K--V--S--D--L--D--R--K--V--S--D-

721  CTGGAGAATGAAGCCAAGAAGCAGGAGGCTGCCATCATGGACTATAACCGAGATATCGAG
241  -L--E--N--E--A--K--K--Q--E--A--A--I--M--D--Y--N--R--D--I--E-
```

781   GAGATCATGAAGGACATTCGCAATCTGGAGGACATCAGGAAGACCTTACCATCTGGCTGC
261   -E--I--M--K--D--I--R--N--L--E--D--I--R--K--T--L--P--S--G--C-

841   TTCAACACCCCGTCCATTGAAAAGCCCTAG
281   -F--N--T--P--S--I--E--K--P--*-

Table S1: qRT-PCR Primer Sets

Gene	Forward Sequence	Reverse Sequence
T	ACCCAGTTCATAGCGGTGAC	CATTGGGAGTACCCAGGTTG
TBX6	AGCCTGTGTCTTTCCATCGT	AGGCTGTCACGGAGATGAAT
PAX3	AGGAAGGAGGCAGAGGAAAG	CAGCTGTTCTGCTGTGAAGG
MEOX1	GAGATTGCGGTAAACCTGGA	GAACTTGGAGAGGCTGTGGA
SIX1	AGTTCTCGCCTCACAACCAC	ACACCCCTCGACTTCTCCTT
MYF5	TCACCTCCTCAGAGCAACCT	GGAACTAGAAGCCCCTGGAG
MYOD1	ACGTGAGGACGAGCATGTG	GTGCAGCGCTTGAGTGTCT
MYOG	TGGGCGTGTAAGGTGTGTAA	CGATGTACTGGATGGCACTG
MSGN1	GGCACCAAAGTCAGGATGTC	GTCTGTGAGTTCCCCGATGT
DMRT2	GAACCACCAAGCAAGGACTTC	CCCAGACCCTGAATACTGCAT
PARAXIS	TCCTGGAGAGCTGTGAGGAT	CACACCCTGTCACCAACAGT
MHC3	GCAGATTGAGCTGGAAAAGG	TCAGCTGCTCGATCTCTTCA
HOXA1	GAGACCCAAGTGAAGATCTGGTT	CCTTCTCGTCGTTTCCTGGCG
HOXB1	GAAACACAGGTCAAGATTTGGTT	GGAAGCCCCATTGGTGGCTAGGT
HOXB2	GAAAGGCAGGTCAAAGTCTGGTT	GGTCGCAGATGTCCTCCAGGG
HOXB3	GAGCGGCAGATCAAGATCTGGTT	GGTAGTTGGAGGGCAGCGCGTAG
HOXB8	GAGAGACAGGTCAAAATCTGGTT	CTTGTCGCCCTTCTGCGCGTC
HOXB9	GAGAGACAAGTCAAAATCTGGTT	GCACTGGCTTTGCAGTCGTCACAT

Table S2: Antibody list and dilution ratio

1st Antibody	Host	Cat. No	Dilution	Company
Myoshin Heavy chain (MHC) MF20	Mouse mono	14-6503	1:800	eBioscience
MyoD1	Rabbit mono	Ab133627	1:500	Abcam
10E4	Mouse mono	370255	1:100	amsbio
Phospho-p44/42 MAPK (ERK1/2)	Rabbit Poly	#9101	1:1000	CST
p44/42 MAPK (ERK1/2)	Rabbit Poly	#9102	1:1000	CST
T	Goat Poly	AF2085	1:100	R&D
TBX6	Goat Ploy	AF4744	1:100	R&D
Antibodies for FACS	Host	Cat. No	Dilution	Company
DLL1-APC	Mouse mono	FAB1818A	1:200	R&D
2nd Antibody			Dilution	Company
Alexa Fluor 568 conjugated goat-anti-mouse IgG		A11031	1:500	ThermoFisher
Alexa Fluor 488 conjugated goat-anti-rabbit IgG		A11034	1:500	ThermoFisher
Alexa Fluor 568 conjugated donkey-anti-goat IgG		A11057	1:500	ThermoFisher
Alexa Fluor 488 conjugated donkey-anti-goat IgG		A11055	1:500	ThermoFisher
Alexa Fluor 488 conjugated goat-anti-mouse IgM,		A21042	1:500	ThermoFisher

Table S3: siRNA sequences used in gene knockdown.

siRNA Target Gene Symbol	Product line	siRNA ID/Cat. No.	Antisence(5'->3')
GAPDH	Silencer™ Select	4390849	
HOXA1	Silencer™ Select	s6753	UAAGGCGCACUGAAGUUCUgt
HOXA1	Silencer™ Select	s6752	AGUUGUAAUCCUAUGGUCCga
HOXB3	Silencer™ Select	s6794	UAGCGGUUAAAAUGGAACUcc
HOXB3	Silencer™ Select	s6796	UGCCUCGACUCUUUCAUCCag

Table S4: Summary of major developmental signaling pathway modulators used in this study; related to Experimental Procedures.

Item name	Description	Company	Cat. No	Con.
AZD4547	FGFR inhibitor	Cellagen Technology	CET-C2454-5S-5	1 μM
BGJ398	FGFR inhibitor	Selleckchem	S2183	1 μM
PD166866	FGFR inhibitor	Sigma	PZ0114	1 μM
PD173074	FGFR inhibitor	Wako	511-94231	1 μM
Wnt-C59	WNT inhibitor	Cellagen Technology	CET-C7641-2S-2	1 μM
DKK1	WNT inhibitor	R&D Systems	5439-DK-010	1 μM
DMH1	BMP inhibitors	Wako	Oct-17	1 μM
LDN193189	BMP inhibitors	ReproCell	04-0074	1 μM
SB505124	TGFβ inhibitor	Wako	Oct-63	1 μM
CP868596	PDGFR inhibitor	CEM	CS-0566	1 μM
Sunitinib Malate	PDGFR inhibitor	Cayman	13159	1 μM
PIK90	PIK inhibitor	Calbiochem	528117-5MGCN	1 μM
PD0325901	ERK inhibitor	Wako	518-91701	1 μM
U73122	PLC inhibitor	Selleckchem	S8011	10 μM

Table S5: Primers for bFGF cloning and mutagenesis.

Primer	Sequence (5'- 3')
pENTR1A-bFGF-FW	GGCTTTAAAGGAACCCCCGCCTTGCCCGAGGAT
pENTR1A-bFGF-RV	AAGCTGGGTCTAGATGCTCTTAGCAGACATTGGA
bFGF-K125E-FW	GCAGTATGAACTTGGATCCAAAACAG
bFGF-K125E-RV	CCAAGTTCATACTGCCCAGTTCGTTT
pMAL-c5X-bFGF-FW	ATGGGCGGCCGCGATCCCGCCTTGCCCGAGGAT
pMAL-c5X-bFGF-RV	GGATCCGTCGACGATTCAGCTCTTAGCAGACATTG
